# Assessment of cytotoxicity and genomic instability induced by coal-derived nanoparticles in V79 and HaCaT Cells

**DOI:** 10.1007/s00204-026-04316-z

**Published:** 2026-04-28

**Authors:** Alvaro Miranda-Guevara, Wilner Martínez-López, Grethel León-Mejía, Ornella Fiorillo Moreno, Leonardo Pacheco-Londoño, Julian Rodríguez Tapia, Fredy Jose Torres Cantillo, Maria Fernanda Palma, Paola Hernández, Pablo Liddle, Burix Mechoso, Miriam López, Laura Lafon-Hughes, Lihuén Villarreal, Juan C. Benech, Renato Puga, Jose Eduardo Vargas, Pedro Fragoso-Castilla, Milton Quintana-Sosa, Juliana da Silva, João Antonio Pêgas Henriques, Antonio Acosta-Hoyos

**Affiliations:** 1 Facultad de Ciencias Básicas, Centro de Investigaciones en Ciencias de La Vida (CICV), Universidad Simón Bolívar, 080002 Barranquilla, Colombia; 2 Departamento de Genética, Instituto de Investigaciones Biológicas Clemente Estable, Ministerio de Educación y Cultura, Montevideo, Uruguay; 3 Servicio de Biodosimetría, Instituto de Investigaciones Biológicas Clemente Estable, Ministerio de Educación y Cultura, Montevideo, Uruguay; 4 Clínica Iberoamerica, Barranquilla, Colombia; 5 Clínica El Carmen, Barranquilla, Colombia; 6 Laboratorio de Señalización Celular y Nanobiología, Instituto de Investigaciones Biológicas Clemente Estable, Ministerio de Educación y Cultura, Montevideo, Uruguay; 7 Plataforma de Microscopía de Fuerza Atómica, Instituto de Investigaciones Biológicas Clemente Estable, Montevideo, Uruguay; 8 Biophysical Chemistry Group, Biological Sciences Department, Centro Universitario Regional Litoral Norte (CENUR-LN), Universidad de La República (UdelaR), Salto, Uruguay; 9 Hospital Israelita Albert Einstein, São Paulo, SP Brazil; 10 Departamento de Biologia Celular, Universidade Federal do Paraná, Curitiba, Brazil; 11 Grupo de Investigación Parasitología Agroecología Milenio, Programa de Microbiología - Universidad Popular del Cesar, Valledupar, Colombia; 12 Programa de Pós-Graduação Em Genética E Biologia Molecular (PPGBM), Universidade Federal Do Rio Grande Do Sul (UFRGS), Porto Alegre, RS Brazil; 13 Laboratory of Genetic Toxicology, La Salle University (UniLaSalle) and Lutheran University of Brazil (ULBRA), Canoas, RS Brazil; 14 Programa de Pós-Graduação Em Biotecnologia E Em Ciências Médicas, Universidade Do Vale Do Taquari - UNIVATES, Lajeado, RS Brazil

**Keywords:** Coal Nanoparticles, Cytotoxicity, Genotoxicity, Oxidative damage, Cell death

## Abstract

Coal is a major global energy resource, but its extraction raises significant environmental and health concerns. Mining activities release large amounts of particulate matter, including nanoparticles (NPs), into the environment. These NPs are hazardous due to their content of polycyclic aromatic hydrocarbons (PAHs), metals, oxides, and other compounds capable of disrupting cellular and molecular processes. This study evaluated the cytotoxic and genotoxic effects of coal-derived NPs on V79 and HaCaT cell lines, focusing on their impact on DNA stability and the mechanisms responsible for cellular damage. NPs were isolated using an acid-based separation method and applied to cells at concentrations of 50, 150, and 300 μg/mL. Atomic force microscopy (AFM) provided topographical characterization, while dynamic light scattering (DLS) confirmed their tendency to agglomerate. Scanning electron microscopy coupled with energy-dispersive X-ray spectroscopy (SEM-EDS) confirmed NP morphology and elemental composition, including carbon, oxygen, iron, calcium, silicon, aluminum, and copper. Cytotoxicity was assessed using resazurin and sulforhodamine B assays, and genotoxicity was evaluated using the comet assay, micronucleus test, and γH2AX immunostaining. Results showed a clear dose-dependent effect, with coal NPs inducing genomic instability and increased cell mortality, mainly through apoptosis. These findings highlight the importance of characterizing coal-derived NPs to better assess their environmental and health risks, particularly regarding respiratory diseases.

**Supplementary Information:**

The online version contains supplementary material available at 10.1007/s00204-026-04316-z.

## Introduction

Coal is an abundant source of energy and continues to play a crucial role in the global economy. However, coal mining activities have led to a wide range of environmental and social impacts. These include the generation of mining waste, deforestation, acid mine drainage, elevated noise levels, contamination of local streams and wetlands, and soil degradation (Morrice and Colagiuri, 2013; Rouhani et al. 2023).

One of the major environmental consequences of coal mining is the release of particulate matter (PM) into the atmosphere (Sultana et al. 2021). This PM includes respirable fractions that can be inhaled by nearby populations and deposited deep in the respiratory tract, potentially causing serious health effects (Zierold et al. 2020). Airborne particles are categorized by their aerodynamic diameter, with smaller particles being more likely to penetrate the distal regions of the lungs (Xu et al., 2022)

In recent years, nanoparticles (NPs) typically ranging from 1 to 100 nm and their aggregates or agglomerates have drawn growing attention due to their potential health implications. Research suggests that nanoscale particles pose a greater risk to human health because of their distinctive properties, such as a high surface area-to-volume or mass ratio. These features enhance their deposition in the lungs and contribute to distinct and often more severe toxicological responses compared to larger particles at the same mass concentration (Xuan et al., 2023).

Concerns regarding the potential adverse health effects of nanoparticle (NP) exposure have intensified over the past decade (P. Zhang et al. 2023), driven by numerous in vitro and in vivo toxicological studies in animals and cell cultures. These studies have demonstrated that NPs can more readily translocate to secondary organs, evade effective clearance by macrophages, travel along sensory neuron axons, and access intracellular structures such as mitochondria and nuclei (Vargas Buonfiglio et al. 2017).

Despite these findings, there remains a lack of comprehensive information regarding the physicochemical characterization of coal-derived NPs particularly those involving transition metals on their surface as well as their genotoxic potential and the molecular mechanisms underlying disease development.

Therefore, the primary objective of this study was to evaluate the in vitro cytotoxic and genotoxic effects of coal-derived NPs using V79 and HaCaT cell lines, in order to deepen our understanding of their potential biological impacts. Ultimately, these insights may contribute to the formulation of stricter regulatory measures aimed at reducing health risks associated with NP exposure.

## Materials and methods

### Separation of coal NPs

Coal NPs were isolated from coal samples obtained from mines in the Loma-Cesar region, using the acid separation protocol described by He et al. (2021). Briefly, 5 g of coal were suspended in nitric acid and stirred continuously for 24 h. The resulting mixture was centrifuged at 4300 rpm for 40 min, and the supernatant was collected. This supernatant was then subjected to a second centrifugation at 15,000 rpm for 1 h. The final supernatant was subsequently heated at 100 °C for 24 h.

The resulting NPs were resuspended in Dulbecco’s Modified Eagle Medium (DMEM) supplemented with antibiotics but without fetal bovine serum (FBS), at a final concentration of 2 mg/mL. To ensure sterility, the suspension was exposed to ultraviolet (UV) light for 30 min. The final NP preparation was used in all subsequent experiments.

### Dynamic light scattering analysis (DLS Analysis)

The coal NP suspension was sonicated using a 400 W Branson Sonifier S-450D (Branson Ultrasonics Corp., Danbury, CT, USA) equipped with a standard 13 mm disruptor horn (model number: 101-147-037). Sonication was performed for 15 min at 400 W and 10% amplitude to ensure proper dispersion of the nanoparticles.

Following sonication, the samples were analyzed by Dynamic Light Scattering (DLS) to determine their hydrodynamic diameter in the dispersion medium. Measurements were carried out using a Zetasizer Nano ZS (Malvern Instruments Ltd., Malvern, UK), employing a refractive index of 1.59 for the composite material. DLS analysis was conducted on three independent batches to ensure accuracy and reproducibility. The Z-average (Z-Ave), representing the mean hydrodynamic diameter, and the polydispersity index (PdI), indicating the width of the nanoparticle size distribution, were both calculated to evaluate particle size distribution and uniformity.

### Atomic force microscopy (AFM)

Topographic images of the nanoparticles were obtained using atomic force microscopy (AFM; Bioscope Catalyst coupled to an Olympus IX-81 microscope). All AFM measurements were performed in Tapping mode using ScanAsyst-Air tips (Bruker) with a nominal tip radius of 2 nm and a nominal cantilever spring constant of 0.4 N/m.

NP suspensions at varying concentrations were prepared in Milli-Q water, and 10 µL of each suspension was deposited onto a mica-coated glass slide for imaging.

### Scanning electron microscopy and Energy-dispersive X-ray spectroscopy (SEM–EDS) analysis

High-vacuum scanning electron microscopy (SEM) was employed to obtain detailed, high-resolution images of the samples, allowing precise analysis of their morphology and surface topography. A secondary electron detector was used to capture fine surface details.

Analyses were performed using a JEOL JSM 6490 LV microscope, which offers robust imaging capabilities under high-vacuum conditions. For sample preparation, the specimens were mounted onto conductive graphite tape to ensure stability and minimize charging artifacts during imaging. A thin layer of gold was then sputter-coated onto the sample surfaces using a Denton Vacuum DESK IV sputter coater, a critical step to enhance conductivity and improve image quality.

Elemental composition was assessed via energy-dispersive X-ray spectroscopy (EDS), using an INCA PentaFETx3 microprobe (Oxford Instruments). This analysis provided both qualitative and semi-quantitative data on the elemental distribution within the samples, complementing the morphological observations. The combination of SEM and EDS analyses enabled comprehensive structural and compositional characterization of the coal NPs.

### Cell culture

The V79 cell line, derived from Chinese hamster lung fibroblasts, was kindly provided by the Argentine Cell Bank Association (ABAC). The human keratinocyte cell line HaCaT was generously donated by the Oncovirology Laboratory, Department of Microbiology, University of São Paulo. Both cell lines were cultured as monolayers in 25 or 75 cm^2^ cell culture flasks under standard conditions using Dulbecco’s Modified Eagle Medium (DMEM) supplemented with 10% heat-inactivated fetal bovine serum (FBS, Capricorn), 0.2 mg/mL L-glutamine, 100 IU/mL penicillin, and 100 μg/mL streptomycin (Capricorn). Cultures were maintained at 37 °C in a humidified atmosphere containing 5% CO₂. For subculturing or establishing V79 cultures, a 0.05% trypsin-EDTA solution and phosphate-buffered saline (PBS) were used.

### Cytotoxicity assays

V79 and HaCaT cells were seeded in 96-well plates at a density of 8000 cells per well in 100 µL of complete culture medium. Cells were obtained from exponentially growing cultures by washing with PBS, detaching with trypsin for 5 min at 37 °C, neutralizing with complete medium, and counting using a Neubauer chamber. Plates were incubated for 24 h at 37 °C in a humidified atmosphere with 5% CO₂ prior to treatment.

Coal NPs were dissolved in DMEM to prepare a stock solution (1 mg of NPs in 500 µL of medium) and subsequently diluted to final concentrations ranging from 12.5 to 400 µg/mL. After removing the medium from the wells, 100 µL of each NP dilution was added in triplicate. Controls included untreated cells as the negative control and cells treated with 250 µM hydrogen peroxide for 1 h as the positive control. Plates were incubated for 24, 48, and 72 h at 37 °C in a 5% CO₂ atmosphere.

Cytotoxicity was assessed using a tandem *resazurin (RZ) and sulforhodamine B (SRB) assays*. For the RZ assay, a 0.025 mg/mL working solution was prepared by diluting 250 µL of a 1 mg/mL resazurin stock solution in 10 mL of PBS. After removing the culture medium, 100 µL of the resazurin solution was added to each well, with PBS-only wells used as blank controls. Plates were incubated for 4 h at 37 °C in the dark, and fluorescence was measured at an excitation wavelength of 530 nm and an emission wavelength of 590 nm using a Varioskan Flash microplate reader.

For the SRB assay, cells were fixed with pre-chilled 10% trichloroacetic acid for 1 h at 4 °C, followed by drying at 37 °C. Cells were then stained with 0.5% SRB in 1% acetic acid for 1 h at room temperature. Excess dye was removed by three quick washes with 1% acetic acid, and plates were dried again at 37 °C. Bound dye was solubilized by adding 100 µL of 10 mM Tris base (pH 10.5) to each well and shaking for 5 min. Absorbance was measured at 570 nm and 600 nm using the Varioskan Flash reader. Results were expressed as relative cytotoxicity.

### Genotoxicity analysis

#### Alkaline standard comet assay

The alkaline comet assay was performed according to the method of Singh et al. (1988), with slight modifications. Briefly, 3 × 10^5^ V79 cells were seeded into each well of 12-well plates. After 24 h of incubation, the cells were exposed for 3 h to coal and coal fly ash, as well as to a negative control (FBS-free medium), and for 2 h to a positive control (400 µM H₂O₂). Following exposure, cells were trypsinized and resuspended in complete medium.

Twenty microliters of the resulting cell suspension were mixed with 90 μL of 0.75% low-melting-point agarose (LMP) and immediately placed onto microscope slides previously coated with 1.5% normal-melting-point agarose (NMP), prepared at 60 °C. After solidification under a coverslip (which was subsequently removed), the slides were immersed overnight in a lysis solution (2.5 M NaCl, 100 mM EDTA, 10 mM Tris, pH 10.0–10.5), supplemented with 1% freshly added Triton X-100 and 10% DMSO, at 4 °C in the dark.

Next, the slides were placed in an alkaline electrophoresis buffer (300 mM NaOH, 1 mM EDTA, pH > 13) for 30 min at 4 °C to allow DNA unwinding. Electrophoresis was then carried out at 25 V and 300 mA for 30 min. Following electrophoresis, slides were neutralized by three 5-min washes in 0.4 M Tris buffer (pH 7.5).

DNA was stained by adding 30 µL of SYBR Safe (10 μg/mL) to each slide, followed by coverslip placement. Comet images were analyzed using OpenComet software. For each treatment and control, a minimum of 50 nuclei per slide were evaluated (two slides per condition). DNA damage was quantified using the percentage of DNA in the comet tail (% Tail DNA) as the primary parameter.

#### Comet assay modified

Oxidative DNA damage was evaluated using a modified version of the comet assay incorporating DNA repair enzymes. In this approach, slides were treated with the enzymes formamidopyrimidine-DNA glycosylase (FPG) and endonuclease III (ENDO III), which introduce strand breaks at sites containing oxidatively damaged bases. FPG recognizes and excises oxidized purines, such as 8-oxoguanine and ring-opened purines (formamidopyrimidines, Fapy), while ENDO III targets oxidized pyrimidines (Collins, 2019; Azqueta et al., 2019).

The modified assay follows the same procedure as the standard alkaline comet assay up to the cell lysis step. After lysis, slides were removed from the lysis solution, rinsed three times with enzyme buffer (40 mM HEPES, 100 mM KCl, 0.5 mM EDTA, 0.2 mg/mL bovine serum albumin; pH 8.0), gently drained, and incubated at 37 °C for 45 min in enzyme buffer containing 60 μL of FPG (1 μg/mL) and ENDO III (1 μg/mL).

Subsequent steps, including DNA unwinding, electrophoresis, and staining, were performed as described for the standard comet assay.

#### Micronucleus test with cytokinesis block (CBMN)

To evaluate the potential genotoxicity of coal NPs, 1.5 × 10^5^ cells per well were seeded into 12-well plates. After 24 h of incubation, cells were exposed either to coal NPs for 24 h or to H₂O₂ (400 µM for 2 h), which served as a positive control. Six hours after the initial treatment, cytochalasin B (3 µg/mL; Sigma-Aldrich, USA) was added to the culture medium, and cells were incubated for an additional 18 h at 37 °C in a humidified atmosphere with 5% CO₂.

Following incubation, cells were trypsinized and centrifuged at 1000 rpm for 5 min at room temperature. The supernatant was discarded, and the cell pellet was treated with cold hypotonic potassium chloride solution (75 mM) for 5 min. Cells were then fixed with a methanol–acetic acid solution (3:1), centrifuged again, and subsequently fixed with pure methanol containing 1% acetic acid. Finally, the cells were spread onto pre-chilled microscope slides.

Cell nuclei were stained with propidium iodide (PI, 20 µg/mL) and visualized under a fluorescence microscope (Zeiss, Axioplan MOT II, Germany). For micronucleus (MN) analysis, 1000 binucleated cells per experimental condition were scored following the criteria established by Fenech (2007). All images were analyzed using MNScoreX software (Metasystems, Altlußheim, Germany).

#### γH2AX immunocytochemistry assay

DNA damage in HaCaT and V79 cells exposed to coal NPs for 24 h was evaluated by quantifying the mean fluorescence intensity (MFI) of γH2AX foci, following the methodology described by Pérez et al. (2019). Briefly, 5 × 10^5^ cells per well were seeded into 6-well plates and incubated for 24 h at 37 °C in a humidified atmosphere with 5% CO₂ to allow cell adherence. Cells were then treated with coal NPs at concentrations of 50, 150, and 300 µg/mL for 48 h. A positive control was included, consisting of cells exposed to H₂O₂ (400 µM) for 1 h.

Following treatment, cells were fixed with 4% paraformaldehyde for 15 min and permeabilized using a blocking buffer containing 0.05% Triton X-100 and 1% BSA in PBS for 40 min at room temperature. Permeabilized cells were incubated overnight at 4 °C with an anti-γH2AX antibody (1:500, ab26350; Abcam, Cambridge, MA, USA). After washing, an Alexa Fluor 488-conjugated secondary antibody (1:1000, goat anti-mouse; Invitrogen, Thermo Fisher Scientific, Waltham, MA, USA) was applied for 45 min at room temperature. DNA was counterstained with DAPI (300 nM; Invitrogen), and slides were mounted using ProLong Glass Antifade Mountant (Invitrogen, Thermo Fisher Scientific, USA).

Fluorescence imaging was performed using a Zeiss Axioplan MOT II fluorescence microscope, and γH2AX foci intensity was analyzed with MetaCyte software (Metasystems, Altlußheim, Germany).

### Analysis of cell death by triple staining

Cells were seeded in 6-well plates at a density of 5 × 10^5^ cells per well and incubated for 24 h in a humidified atmosphere containing 5% CO₂ at 37 °C. Subsequently, the cells were treated with coal nanoparticles (NPs) at concentrations of 50, 150, and 300 µg/mL for 48 h. Positive controls were exposed to 400 µM hydrogen peroxide (H₂O₂) for 1 h.

A fluorescent staining solution was prepared containing Hoechst 33,342 (2 µg/mL), fluorescein diacetate (FDA, 15 µg/mL), and propidium iodide (PI, 5 µg/mL) in sterile water maintained at 4 °C.

Following treatment, the cells were washed twice with PBS, trypsinized, neutralized with culture medium, and centrifuged at 3500 rpm for 5 min. The supernatant was discarded, and 2 × 10⁶ cells were resuspended in 400 µL of PBS. Then, 8 µL of the staining solution was added to the cell suspension. After a brief incubation, 7 µL of the suspension were used to prepare slides.

A total of 300 live cells were analyzed within 20 min using a fluorescence microscope (Zeiss, Axioplan MOT II, Germany).

### Melting analysis

A real-time PCR amplification of a DNA segment from Human Papillomavirus type 51 (HPV 51) was performed using primers targeting the L1 region of the viral genome, as described by Tsakogiannis et al. (2015). The primers used were HPV-51L1 F (5´–3´: CCAATACCTAAAACCTCAAC) and HPV-51L1 R (5´–3´: ACAACCCCACACCAACCTA). Each PCR reaction was carried out in a final volume of 20 µL.

Amplification conditions included an initial denaturation at 94 °C for 4 min, followed by 40 cycles of denaturation at 94 °C for 1 min, annealing at 50 °C for 1 min, and extension at 72 °C for 1 min, with a final extension step at 72 °C for 10 min.

Melting curve analysis using SYBR™ Green was performed by increasing the temperature from 60 °C to 94 °C at a rate of 0.3 °C per second. Melting temperature data were used to generate a derivative plot of fluorescence intensity (1stD(RFU)) versus temperature.

The amplified HPV 51 product was purified using the PureLink™ PCR Purification Kit (Invitrogen™), and DNA concentration was quantified using a NanoDrop™ One UV spectrophotometer (Thermo Scientific™).

To verify the size and integrity of the purified product, electrophoresis was conducted on a 2% agarose gel in 1 × TAE buffer (Tris-acetate-EDTA, Promega). PCR products were stained with SYBR™ Safe DNA Gel Stain (Invitrogen™), and a 5 µL sample was mixed with 2 µL of Gel Loading Dye (New England Biolabs) and loaded onto the gel. A 100 bp DNA ladder (New England Biolabs) was used as a molecular weight marker. Electrophoresis was run at 70 V for 105 min, and gel images were captured using a BIO-RAD Gel Doc™ XR + Imaging System with Molecular Imager® software.

To investigate the interaction of the purified DNA fragment with graphene and coal NPs, dual melting analysis was performed. The experimental conditions included: (i) purified HPV 51 DNA, (ii) DNA mixed with the coal NP solution, (iii) the coal NP solution with nuclease-free water (negative control), and (iv) nuclease-free water alone (positive control). Each preparation included 2 µL of SYBR™ Safe. Samples were analyzed using real-time PCR protocols, and results were interpreted according to the methodology described by Ivask et al. (2015).

#### Systems biology analysis

Input data for predicting a protein–protein interaction (PPI) network involving different types of regulated cell death (apoptosis, autophagy, ferroptosis, necroptosis, and pyroptosis), as well as various DNA repair pathways, were retrieved from the Kyoto Encyclopedia of Genes and Genomes (KEGG) database (Kanehisa et al. 2023). To construct a chemical–protein (CP)–PPI network integrating KEGG data with metal elements identified by SEM-EDS analysis of coal nanoparticles from the mines, the metasearch engine STITCH 5.0 (Search Tool for Interactions of CHemicals; Szklarczyk et al. 2016) was employed.

The network was constructed using the criteria “experiments,” “databases,” and “co-expression,” with an interaction confidence score threshold of 0.4. Unconnected nodes were excluded from the analysis. Degree and betweenness centrality metrics were calculated using Cytoscape software, applying the CentiScaPe 2.2 plugin (Scardoni et al., 2009). Nodes with degree values above the network average were classified as hubs (H), while nodes with betweenness centrality values exceeding the average were considered bottlenecks (B).

The constitutive gene expression profile of the HaCaT cell line was analyzed using the rank product (RP) method, which identifies differentially expressed genes by evaluating rank distributions across replicate experiments. Expression data replicates were obtained from the GEO database (accession number GSE143521). RP analysis was performed with a significance threshold of *p* ≤ 0.01 and *q* ≤ 0.05.

Additionally, Venn diagrams were generated using the online tool available at http://bioinformatics.psb.ugent.be/webtools/Venn/.

#### Statistical analysis

Each experiment was performed in triplicate, and data were expressed as mean ± standard error of the mean (SEM). Each experimental group was compared to the untreated control group using one-way analysis of variance (ANOVA), followed by Tukey’s post hoc test to evaluate pairwise differences. Statistical analyses were performed using GraphPad Prism version 5.0 (GraphPad Inc., San Diego, CA, USA). A *p*-value of less than 0.05 was considered statistically significant, indicating meaningful differences between groups.

## Results

### Characterization of coal NPs

#### Dynamic light scattering (DLS) analysis

Following the isolation of coal NPs extracted from mines located in La Loma, Cesar, their hydrodynamic size was determined by dynamic light scattering (DLS) analysis. The DLS analysis revealed a highly polydisperse size distribution, with approximately 16.57% of the particles exhibiting sizes close to 40 nm (Figure S1). However, the overall population spans a much broader range, reaching values above 500 nm, indicating aggregation and heterogeneity within the sample. This finding provides essential information on the size distribution of the particles and contributes to a more comprehensive understanding of their physical characteristics, particularly in relation to potential interactions with biological systems.

#### Atomic force microscopy (AFM)

**Fig. 1:**
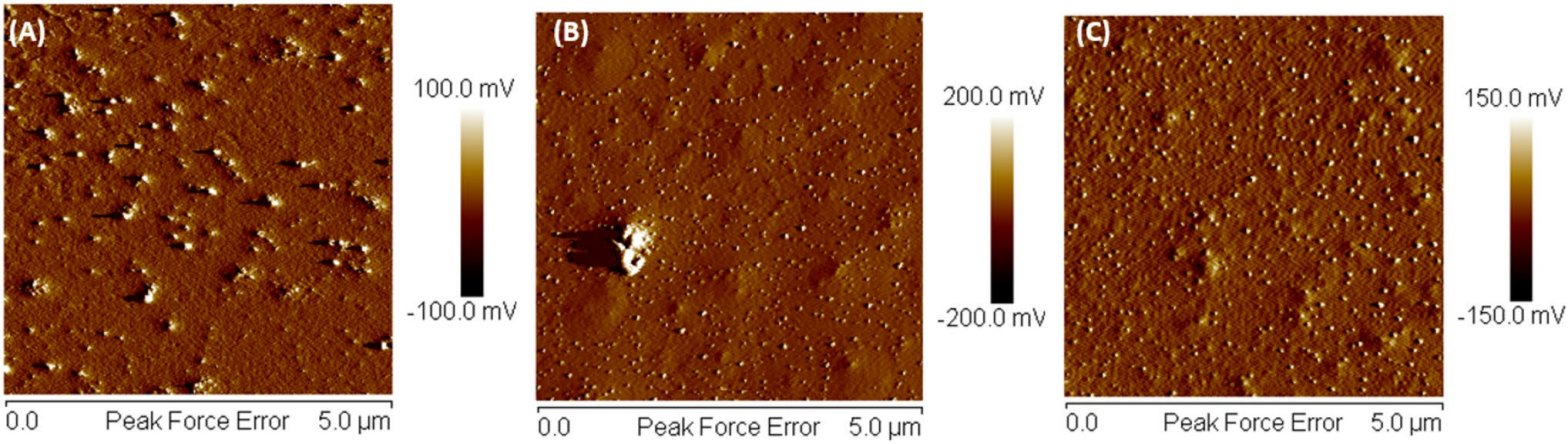
Atomic Force Microscopy (AFM) images of coal NPs at different concentrations **A** 50 μg/mL; **B** 150 μg/mL; **C** 300 μg/mL

The topography of the coal NPs was analyzed using atomic force microscopy (AFM) (Fig. 1A, B, C). The coal NPs exhibited a predominantly spherical morphology, although some particles presented amorphous features. A tendency to form clusters was observed, likely due to their high surface energy, as illustrated in Fig. 1B.

The number of coal NPs detected by AFM increased proportionally with the applied concentrations (50 µg/mL, 150 µg/mL, and 300 µg/mL). Particle sizes ranged from 22 to 44 nm, consistent with the measurements obtained by dynamic light scattering (DLS) (Figure S1). This agglomeration behavior highlights the importance of characterizing NP topography and spatial distribution, as these parameters are critical for understanding their potential biological effects.

#### Scanning electron microscopy with Energy-dispersive X-ray spectroscopy (SEM–EDS)

**Fig. 2:**
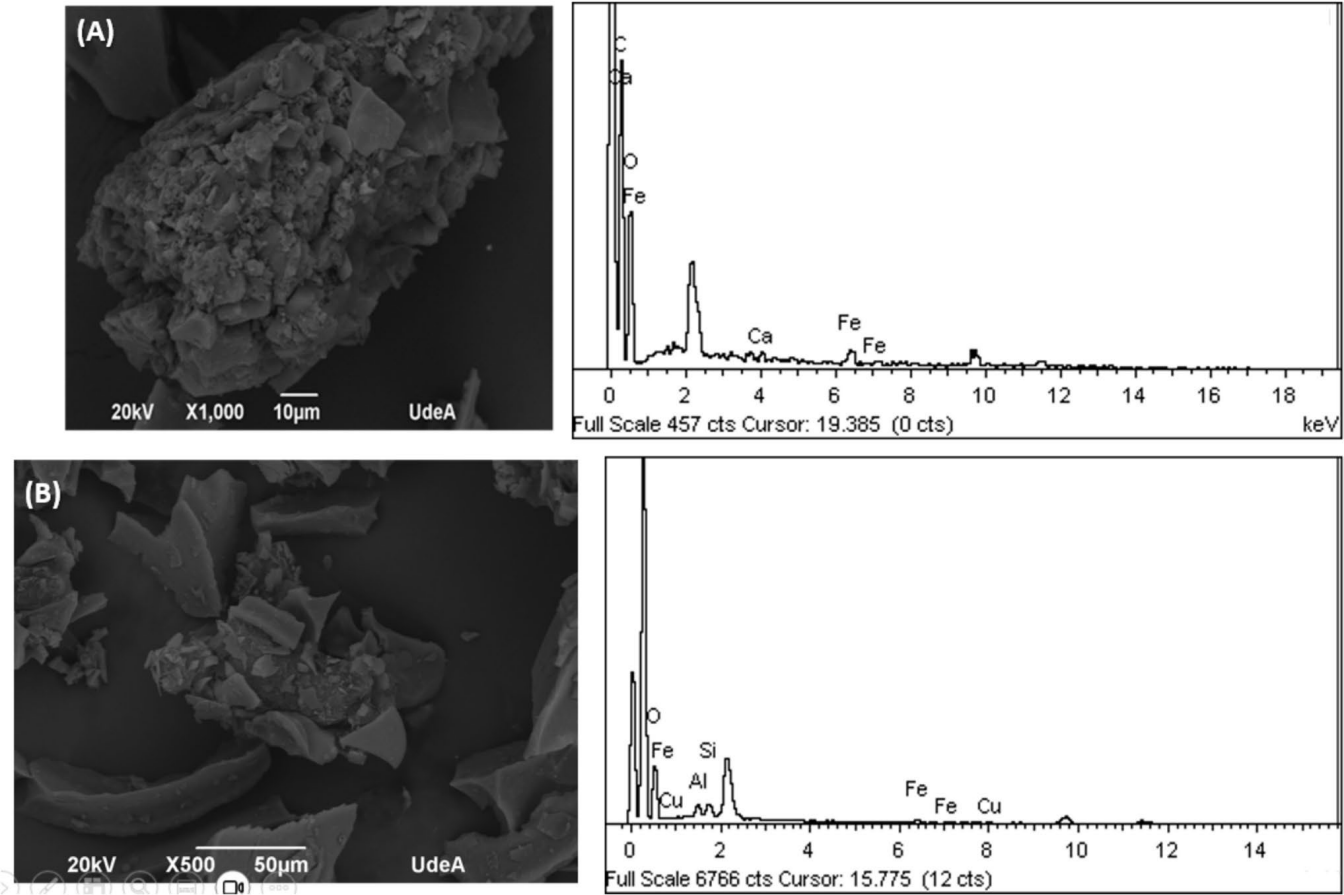
SEM–EDS analysis of coal NPs from the mines of La Loma-Cesar. The Figure A and B show the irregular shape of the NPs and next the EDS compositional spectrum

The analysis of coal NPs using scanning electron microscopy coupled with energy-dispersive X-ray spectroscopy (SEM-EDS) (Fig. 2A, B) revealed an amorphous morphology and the formation of aggregates with variable chemical compositions. Elements such as carbon (C), oxygen (O), iron (Fe), calcium (Ca), silicon (Si), aluminum (Al), and copper (Cu) were identified.

### Cytotoxicity analysis

#### Resazurin and sulforhodamine B Assays

**Fig. 3:**
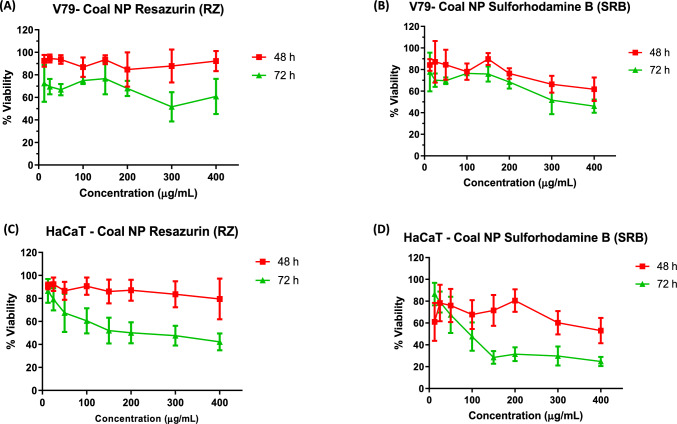
Cell viability analysis using different assays **A** Resazurin and **B** Sulforhodamine B at different exposure periods in V79 cells. **C** Resazurin and **D** Sulforhodamine B at different exposure periods in HaCaT cells. Experiments conducted in triplicate

The cytotoxic effects of coal NPs on V79 and HaCaT cell lines were assessed using resazurin-based fluorometric and sulforhodamine B-based colorimetric assays. Figures 3A, B show the survival rates of V79 cells exposed to varying concentrations of coal NPs, demonstrating significant cytotoxicity after 48 h of exposure. Similarly, Figs. 3C, 3D depict the viability trends in HaCaT cells, which also exhibited a marked decrease in viability following 48 h of treatment with coal NPs. At 72 h, a very pronounced and relevant decrease in cell viability was observed, indicating that the 48-h period was more suitable for analysis, as the 72-h exposure led to a drastic reduction in viability. In both cell types, the most significant effects were observed at concentrations close to 300 µg/mL. Based on these findings, we selected concentrations of 50, 150, and 300 µg/mL for subsequent genotoxicity analyses.

### Genotoxicity analysis

#### Alkaline and lesion-specific endonuclease-modified comet assay

To evaluate the genotoxicity of coal NPs, both the standard comet assay and a modified version using enzymes were performed. V79 and HaCaT cells were exposed to NP concentrations determined through cytotoxicity assays for 48 h.

**Fig. 4:**
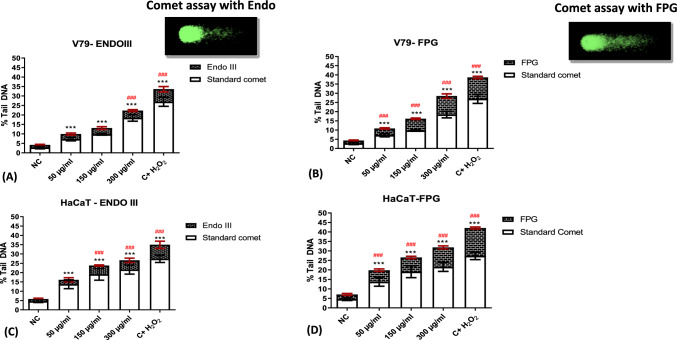
Percentage of DNA in the tail (% Tail DNA) in the alkaline comet assay (white) and percentage of oxidative DNA damage in the modified comet assay (dotted) after treatment with ENDO III and FPG enzymes. In A-B ENDO III and FPG enzyme in V79 cells respectively and C-D ENDO III and FPG enzyme in HaCaT cells respectively. *Statistically significant difference compared to the negative control (NC) (*P* < 0.05). #Significant increase in oxidative damage compared to the negative control with ENDO III and FPG (*P* < 0.05). Results are presented as the mean ± standard error

The graphical representation of the % Tail DNA values (Fig. 4) illustrates the relationship between NP concentration and the extent of genetic damage observed in both cell lines. Statistical analysis revealed significant differences in DNA damage levels as NP concentration increased (*P* < 0.05), with the highest values recorded in the positive control with H_2_O_2_.

In V79 cells, using the ENDOIII enzyme (Fig. 4A), a significant increase in oxidative damage was observed only at the highest concentration (300 µg/mL). However, when using the FPG enzyme, the increase in oxidative damage compared to the negative control was significant even at the lowest concentration (50 µg/mL) (Fig. 4B).

In HaCaT cells, the use of ENDO III (Fig. 4C) revealed significant oxidative damage at a concentration of 150 µg/mL. In contrast, with the FPG enzyme (Fig. 4D), significant damage was observed starting at the lowest concentration (50 µg/mL), consistent with the results in V79 cells. These values in HaCaT cells were somewhat more pronounced than those observed in V79 cells.

#### Micronucleus assay

The formation of micronuclei was evaluated after 48 h of exposure of V79 and HaCaT cells to various concentrations of coal NPs (50, 150, and 300 μg/mL). The results showed an increase in the frequency of micronuclei with increasing NP concentrations; however, this response was not strictly proportional, suggesting a nonlinear dose–response pattern commonly observed with nanomaterials. These findings were statistically significant when compared to the negative control group. The highest frequency of micronucleus formation was observed at 300 µg/mL and in the positive control with H₂O₂ (Table 1).

**Table 1:** Frequency of micronuclei-induced by coal NPs in V79 and HaCaT cells

Cell	Groups (µg/mL)		MN
	0		4.0 ± 1.0
V79	50		6.3 ± 1.52***
	150		7.3 ± 2.0***
	300		11.3 ± 2.51***
	400 mM H_2_O_2_		14.6 ± 2.5***
HaCaT	0		3.6 ± 1.5
	50		6.8 ± 1.5***
	150		9.6 ± 1.5***
	300		11.7 ± 2.0***
	400 mM H_2_O_2_		13.3 ± 2.5***

Experiments conducted in triplicate. *Statistically significant difference compared to the negative control. Tukey’s multiple comparison test, P < 0.05

#### Immunostaining using anti-γ-H2AX antibodies

**Fig. 5:**
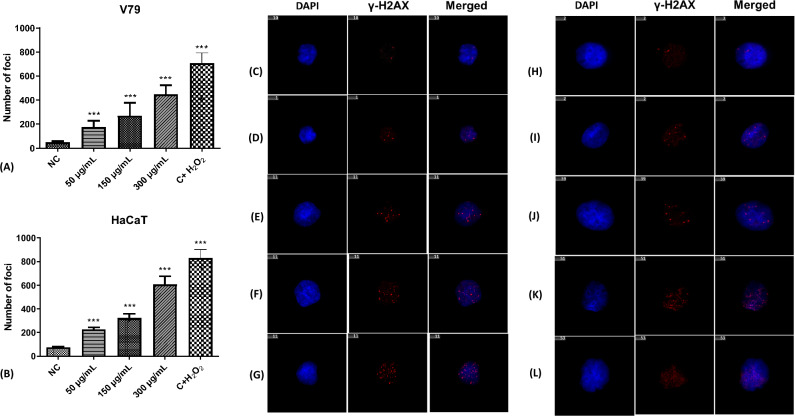
Immunofluorescence of γ-H2AX foci in **A** V79 and **B** HaCaT cells exposed to different concentrations of coal NP (50, 150, and 300 μg/mL). Images revealing the foci in V79 cells: **C** Negative Control (cells cultured only with DMEM medium). **D**, **E**), and **F** Cells treated with coal NPs at 50, 150, and 300 μg/mL, respectively. **G** Cells treated with H_2_O_2_ (400 mM) (positive control). Images revealing the foci in HaCaT cells: **H** Negative Control (cells cultured only with DMEM medium). **I**, **J**, and **K** Cells treated with coal NPs at 50, 150, and 300 μg/mL, respectively. **L** Cells treated with H_2_O_2_ (400 mM) (positive control)

Figure 5 shows a significant increase in the number of γ-H2AX foci in both V79 and HaCaT cells, with a clear dose-dependent relationship. The highest concentrations induced the greatest number of foci, indicating an increased formation of double-strand breaks. This finding is further supported by the representative images shown in Figs. 5C–L for both cell lines.

### Cell death analysis

Cell death analysis was conducted to assess the induction of different types of cell death, including apoptosis and necrosis. Table 2 summarizes the results obtained after exposure to various concentrations of coal NPs, revealing statistically significant increases in cell death. Notably, apoptosis predominated over necrosis in both V79 and HaCaT cell lines. These findings suggest that coal NPs have a substantial impact on cellular homeostasis and can actively trigger programmed cell death pathways.

**Table 2:** Cell death analysis in V79 and HaCat cells exposed to different concentrations of coal NPs

Parameters	NC	H_2_O_2_	50 µg/mL	150 µg/mL	300 µg/mL
V79 cells					
Early apoptosis	0.0 ± 0.0	50.0 ± 1.0***	5.0 ± 0.6***	22.0 ± 1.6***	40.0 ± 1.5***
Late apoptosis	3.0 ± 0.5	99.0 ± 1.4***	3.5 ± 0.4	34.0 ± 0.8***	82.0 ± 2.7***
Necrosis	5.0 ± 1.4	51.0 ± 3.5***	6.0 ± 0.6	19.0 ± 1.5***	31.0 ± 0.6***
HaCaT cells					
Early apoptosis	3.0 ± 0.3	58.0 ± 0.7***	4.0 ± 0.3	40.5 ± 0.3***	57.0 ± 0.5***
Late apoptosis	4.0 ± 0.2	92.0 ± 0.7***	6.0 ± 0.4**	17.0 ± 0.4***	73.0 ± 0.5***
Necrosis	2.0 ± 0.2	65.0 ± 0.4***	10.0 ± 0.4***	13.0 ± 0.4***	29.0 ± 0.6***

Values are expressed as frequency of cells (mean ± standard error). Experiments performed in triplicate. *Statistically significant difference compared to the negative control (NC). Tukey’s multiple comparison test, *P* < 0.05

### DNA interaction with coal NPs determining melting temperature

Using the melting temperature data obtained from the amplification process, a representative graph was generated plotting the first derivative of the fluorescence signal (1stD(RFU)) against temperature. In this graph (Figure S2), the peaks represent the melting temperatures of the HPV fragment. After purification of the amplified HPV-51 material, DNA concentration was determined using a UV absorption spectrophotometer (NanoDrop™ One, Thermo Scientific™), yielding a value of 20 ng/µL based on absorbance at 260 nm. The 260/280 absorbance ratio was 2.0, indicating high purity with minimal protein contamination. Additionally, the 260/230 ratio was 2.1, suggesting negligible contamination by organic compounds or salts. These values confirm that the concentration and purity of the genetic material were optimal. The size and integrity of the purified product were confirmed by agarose gel electrophoresis. Notably, the variation in melting temperature peaks observed in Figure S2 suggests a potential interaction between DNA and coal- or graphene-derived nanoparticles, which may lead to a decrease in melting temperature and reduced double-stranded DNA (dsDNA) stability.

### Human network analysis

A chemical-protein (CP) and protein-protein interaction (PPI) network analysis was conducted to explore potential pathway imbalances induced by inorganic elements present in coal NPs, specifically metal components such as aluminum (Al), calcium (Ca), copper (Cu), iron (Fe), and silicon (Si). A total of 882 genes or proteins related to cell death or DNA repair, along with the identified metals shown in Fig. 2, were used as the initial dataset to construct the CP–PPI network.

Following STITCH analysis, the resulting CP–PPI network consisted of 587 nodes and 6,376 edges (Figure S3a). A subsequent centrality analysis was conducted to identify hub (H) and bottleneck (B) nodes (Figure S3b). The top 10 H-B proteins identified were TP53, UBB, HSP90AA1, BRCA1, PCNA, CASP3, IKBKB, RPS27A, RELA, and TRAF6 (Supplementary Table S4). Notably, Ca emerged as a predominant H-B node, along with Fe and Cu, suggesting that these metals play critical roles in modulating key cellular signaling pathways.

To further investigate this hypothesis, all 110 H-B proteins and their interactions with metals were examined (Figure S3c). Among them, Ca was found to regulate 31 H-B proteins, followed by Fe (9), Cu (7), and Al (1). Based on this analysis, 39 H-B proteins (35.45% of the total) were identified as being directly modulated by metals.

To assess whether this direct modulation influences cell fate, these 39 H-B proteins were analyzed in the context of signaling pathways (Figure S3d). Interestingly, 15 of them were involved in apoptosis pathways, with some also overlapping with autophagy and pyroptosis pathways. This suggests a diverse and complex cellular response potentially triggered by exposure to coal-derived NPs. Finally, to confirm the in vitro cell viability results, we analyzed constitutively upregulated genes in the HaCaT cell line and their overlap with H-B and M-H-B nodes (Figure S3e). A greater number of H-B proteins overlapped with HaCaT expression than with M-H-B nodes, suggesting that coal NP-induced cell death in HaCaT cells may predominantly occur through indirect modulation of key signaling pathways.

## Discussion

Nanoparticles released during coal mining activities can significantly impact surrounding ecosystems (Hendryx et al. 2020). Due to their small size, large surface area, and low density, these NPs can rapidly disperse into the environment, facilitating their transport through air, water, and soil (Rönkkö and Timonen 2019). These physicochemical properties contribute to their wide distribution and increase the likelihood of interactions with biological systems.

In this study, the hydrodynamic characterization of coal-derived NPs revealed an average particle size of 40 nm with a homogeneous distribution. These findings are consistent with previous studies demonstrating that NP size, geometry, and surface characteristics play crucial roles in determining cellular interactions (Dong et al. 2020; Kumah et al. 2023; Sohaebuddin et al. 2010). For example, spherical NPs are generally less efficiently absorbed but tend to exhibit higher toxicity. Moreover, a positive surface charge enhances interactions with cellular membranes, increasing the risk of structural and functional damage (Bruinink et al. 2015; Dong et al. 2020).

AFM and SEM offered complementary morphological information reflecting the different measurement conditions. SEM, performed on dry samples, revealed irregular and angular coal fragments, consistent with the heterogeneous mineral structure of coal. In contrast, AFM analysis was conducted on NPs in suspension, where nanoscale rounded domains became evident, likely due to hydration and colloidal stabilization. Thus, the combined data indicate that coal NPs are irregular aggregates that contain nanoscale substructures, whose morphology varies depending on the physical state of the sample and may influence their bioavailability and toxicological behavior (Augustine et al. 2020; Yuan et al. 2019). In the cytotoxicity assessment, NPs caused a significant reduction in cell viability at concentrations of 300 µg/mL after 48 h in both V79 and HaCaT cell lines, suggesting a dose-dependent effect consistent across different cell types.

The ability of NPs to generate reactive oxygen species (ROS) emerges as a central mechanism of cytotoxicity, as these ROS can damage membranes, proteins, and DNA (Yuan et al. 2019). Additionally, aggregated NPs may facilitate cellular entry via endocytosis, disrupt intracellular processes, and trigger inflammatory responses through cytokine release (De Stefano et al. 2012; Yuan et al. 2019). Furthermore, NPs may access the nucleus through nuclear pores and induce DNA damage, including single- and double-strand breaks, which are part of the initial oxidative DNA damage processing (Sambandam et al. 2015; Wan et al. 2019). In this context, coal NPs may promote intracellular ROS production through the activation of redox-active surface groups and metal contaminants capable of participating in Fenton-like reactions, thereby amplifying oxidative stress and contributing to the genotoxic and cytotoxic outcomes observed in both cell lines.

The genotoxicity assessment of coal NPs, conducted using the micronucleus and comet assay, demonstrated their ability to induce genetic damage in V79 and HaCaT cells in a dose-dependent manner. The use of the FPG enzyme in the comet assay detected significant oxidative damage even at low concentrations (50 µg/mL), highlighting its sensitivity in identifying oxidative lesions in purines. In the micronucleus assay, a significant increase in micronucleus frequency was observed after 48 h of exposure to increasing NP concentrations, reinforcing the correlation between NP dose and genetic damage. Micronuclei, formed by lagging chromosomes or chromosomal fragments, reflect dysregulation in fundamental processes such as DNA replication and repair, ultimately impacting chromosome integrity (Fenech et al. 2020; Krupina et al. 2021; Sambandam et al. 2015).

γ-H2AX analysis revealed a significant increase in positive foci in both exposed cell lines, even at the lowest concentration, indicating an accumulation of DNA double-strand breaks. This finding suggests that NPs interfere with DNA repair mechanisms, exacerbating genomic damage and correlating with the previously described micronucleus formation (Wan et al. 2012; Lee et al. 2019; Redon et al. 2011). Interestingly, triple staining used to assess cell death showed a significant incidence beginning at the lowest NP exposure concentration, which may be associated with an excess of DNA lesions. This damage overload could overwhelm repair mechanisms and activate apoptotic or necrotic pathways to eliminate the compromised cells (León-Mejía et al. 2016; Roos et al. 2016).

The chemical composition of the NPs—including elements such as carbon, oxygen, iron, calcium, aluminum, silicon, and copper—may have contributed to the observed cellular effects. Carbon, in particular, affects adsorption and penetration into cellular membranes, modulating toxicity (Cheng et al. 2009; Géloën et al. 2021). The presence of metals such as iron, aluminum, and copper, along with metalloids like silicon in coal NPs, has significant implications for ROS generation and subsequent cell death (Kessler et al., 2022). Transition metals like iron and copper can participate in redox reactions, such as the Fenton and Haber–Weiss cycles, catalyzing the formation of hydroxyl radicals (·OH), which are highly reactive and damaging to cellular macromolecules, including lipids, proteins, and DNA.

Furthermore, although aluminum and silicon are less reactive than transition metals, they can still induce oxidative stress by disrupting cellular homeostasis and triggering inflammatory responses (Fernandes et al., 2021; Rafieepour et al., 2024). This redox imbalance may activate apoptotic and necrotic pathways, thereby compromising cell viability. In addition to apoptosis and necroptosis, ferroptosis and pyroptosis are also known to be triggered by environmental particulates (Qian et al., 2021; Sagawa et al., 2021; Yue et al., 2023). Systems biology analysis suggests that the apoptosis induced by coal NPs may, at least in part, be attributed to the presence of metals. Although experimental confirmation falls outside the scope of this study, autophagy and pyroptosis could also potentially be involved. Moreover, calcium, iron, and copper associated with coal NPs appear to be strong candidates for explaining the observed cell death. Regardless of the specific mode of cell death, DNA damage seems to play a central role at the network level.

A plausible explanation for the observed genotoxicity is that coal NPs may interact with DNA, either directly or indirectly. Although not all nanoparticle types can enter the nucleus through nuclear pores, some studies have shown that carbon-based NPs are internalized via endocytosis and accumulate in intracellular compartments, sometimes reaching close proximity to the nucleus (Yuan et al. 2019; De Stefano et al. 2012). Additionally, other reports suggest that certain small or surface-functionalized NPs may approach or partially access the perinuclear region, enabling potential contact with DNA without requiring full translocation across the nuclear envelope (Sambandam et al. 2015; Wan et al. 2019). These considerations indicate that, while direct NP–DNA contact is possible under specific physicochemical conditions, it may not represent the predominant mechanism in more complex cellular environments.

Supporting the possibility of NP–DNA interaction, our melting-temperature and real-time PCR analyses revealed a decrease in DNA stability following exposure. This finding aligns with other in vitro studies—typically conducted in low-protein or protein-free media—which report that carbonaceous NPs can adsorb onto DNA molecules, disrupt intermolecular interactions, and induce partial destabilization of the double-stranded structure (Fan et al. 2022; Khramtsov et al. 2018; Safaee et al. 2019). Such alterations have been attributed to non-covalent interactions, including electrostatic forces, van der Waals interactions, and hydrogen bonding between DNA bases and hydroxyl or carbonyl groups on NP surfaces (Li et al. 2019; Nii et al. 2013; Shearer et al. 2017; Zhang et al. 2021).

Nonetheless, considering the strong capacity of coal NPs to induce intracellular ROS, it is equally plausible that the DNA damage observed in this study arises predominantly from indirect oxidative pathways rather than from direct physical contact with DNA. ROS can readily generate single- and double-strand breaks, base modifications, and oxidative lesions independent of nanoparticle entry into the nucleus (Ishibashi et al. 2017; Z. Zhang et al. 2023).

By integrating physicochemical characterization with complementary cytotoxicity and genotoxicity assays, our results indicate that these NPs could interfere with key cellular processes involved in DNA integrity, repair mechanisms, and apoptosis regulation. Elements detected within the NPs, such as calcium, iron, and copper, may play a role in modulating biological responses through their interaction with regulatory proteins, which could amplify the observed effects. Although these observations cannot fully replicate real environmental exposures, they provide preliminary insight into how particulate matter originating from coal extraction might influence cellular processes, particularly in contexts where inhalation of such particles is frequent.

Although our study focuses on coal-derived nanoparticles as environmental contaminants with clear cytotoxic and genotoxic effects, it is important to contextualize these findings within the broader field of nanoparticle research, where other nanomaterials have demonstrated therapeutic potential. For example, several studies have reported that metallic or composite nanoparticles can exert antioxidant, anticancer, neuroprotective, or anti-inflammatory activities (Abdoli et al. 2020; Dou et al. 2021; Han et al. 2020; Li et al. 2020; Yan et al. 2020a, b, c). Gold-decorated magnetic nanocomposites and green-synthesized copper nanoparticles have shown selective cytotoxicity against lung and ovarian cancer cell lines (Dou et al. 2021; Li et al. 2020), while polymer–graphene oxide nanofibers enable controlled drug delivery with minimal toxicity to normal cells (Abdoli et al. 2020). Likewise, copper nanoparticles derived from *Nigella sativa* exhibit neuroprotective properties (Yan et al. 2020a, b, c), and Ag-based magnetic composites reduce apoptosis and inflammation in human lung cells exposed to toxic agents (Han et al. 2020). In contrast, the coal NPs analyzed in our study triggered apoptosis, DNA fragmentation, and genomic instability, reinforcing that their composition drives harmful biological responses rather than therapeutic ones. Nevertheless, these contrasts emphasize the need for a more comprehensive understanding of the toxicity mechanisms of coal-derived nanoparticles, which is essential for accurate risk assessment and for guiding future research on nanoparticle safety.

Overall, this study suggests the importance of further exploring the properties and biological impact of coal-derived NPs to better understand their potential environmental and public health implications, especially in regions affected by mining activities.

## Limitations of the study

Like any experimental work, this study has certain limitations that should be considered when interpreting the results. The use of two immortalized cell lines (V79 and HaCaT) provides a controlled and reproducible system to investigate the effects of coal-derived nanoparticles; however, these models cannot fully reproduce the structural and functional complexity of human tissues or the systemic interactions that occur during prolonged environmental exposure. Likewise, although the elemental composition of the particles was determined through SEM-EDS, the chemical characterization did not include more refined analyses such as PAH profiling or metal speciation that could further influence nanoparticle behavior, reactivity, and toxicity. Despite these constraints, the approaches used here offer a solid and informative foundation, generating valuable preliminary evidence that strengthens the relevance of the findings and provides a robust starting point for future investigations

## Supplementary Information

Below is the link to the electronic supplementary material.

Supplementary file1 (DOCX 1531 KB)

## Data Availability

The datasets generated and/or analyzed during the current study are available from the corresponding author on reasonable request.
