# Supplementary material for: Assessment of cytotoxicity and genomic instability induced by coal-derived nanoparticles in V79 and HaCaT Cells
Source: Arch Toxicol. 2026 Apr 28;100(8):3395–409. doi: 10.1007/s00204-026-04316-z (PMC13379404; doi:10.1007/s00204-026-04316-z)
Supplement: Supplementary file 1 — Supplementary file1 (DOCX 1531 KB) [file 204_2026_4316_MOESM1_ESM.docx]

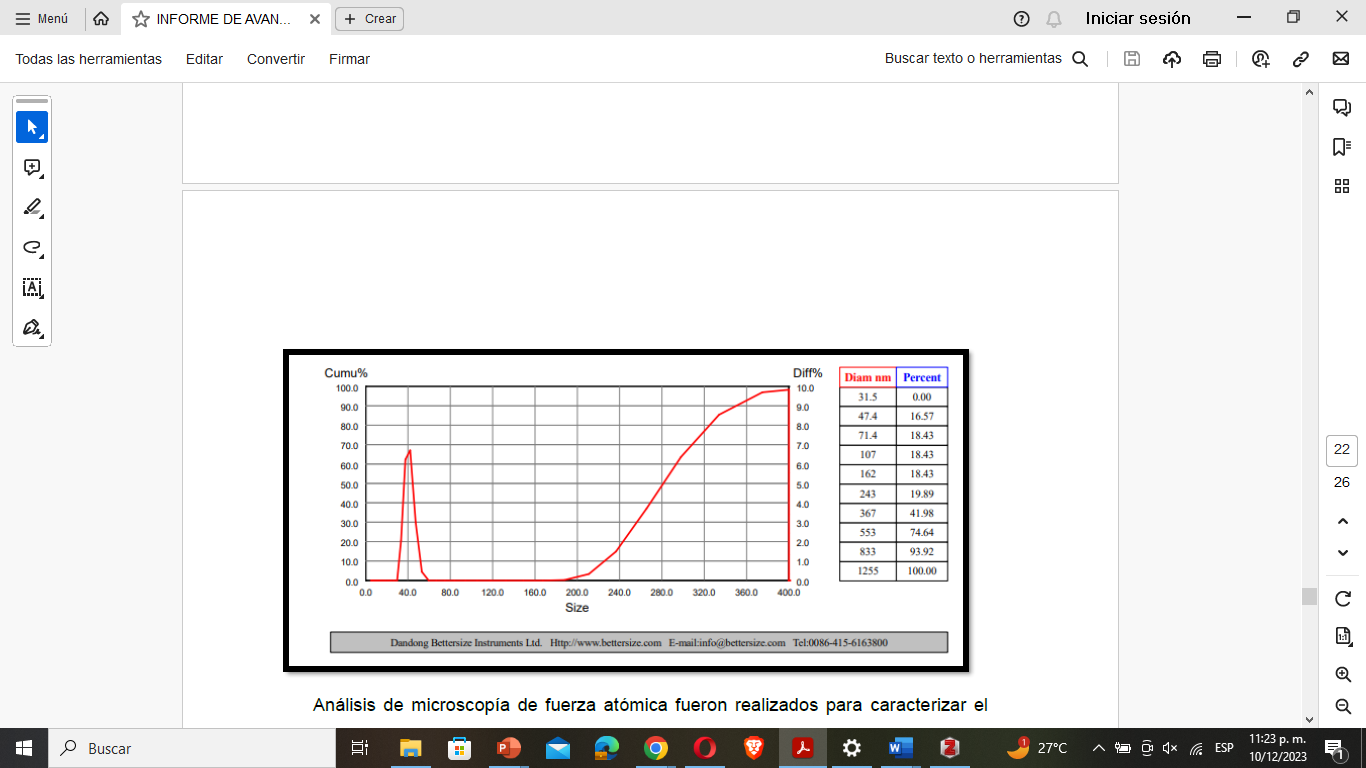


**S1.** Hydrodynamic size of the coal NP analyzed through dynamic light scattering (DLS).


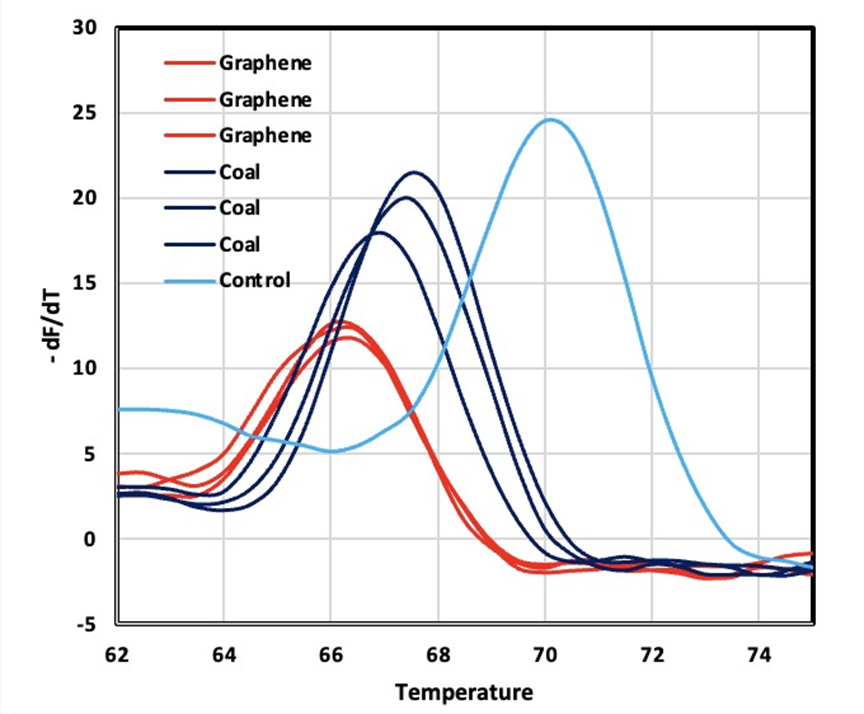


**S2.** Melting temperature (Tm) curve obtained for HPV 51 following the SYBR Green real-time PCR process. Y-axis: First derivative of the fluorescence profile 1stD(RFU); X-axis: Temperature in °C.


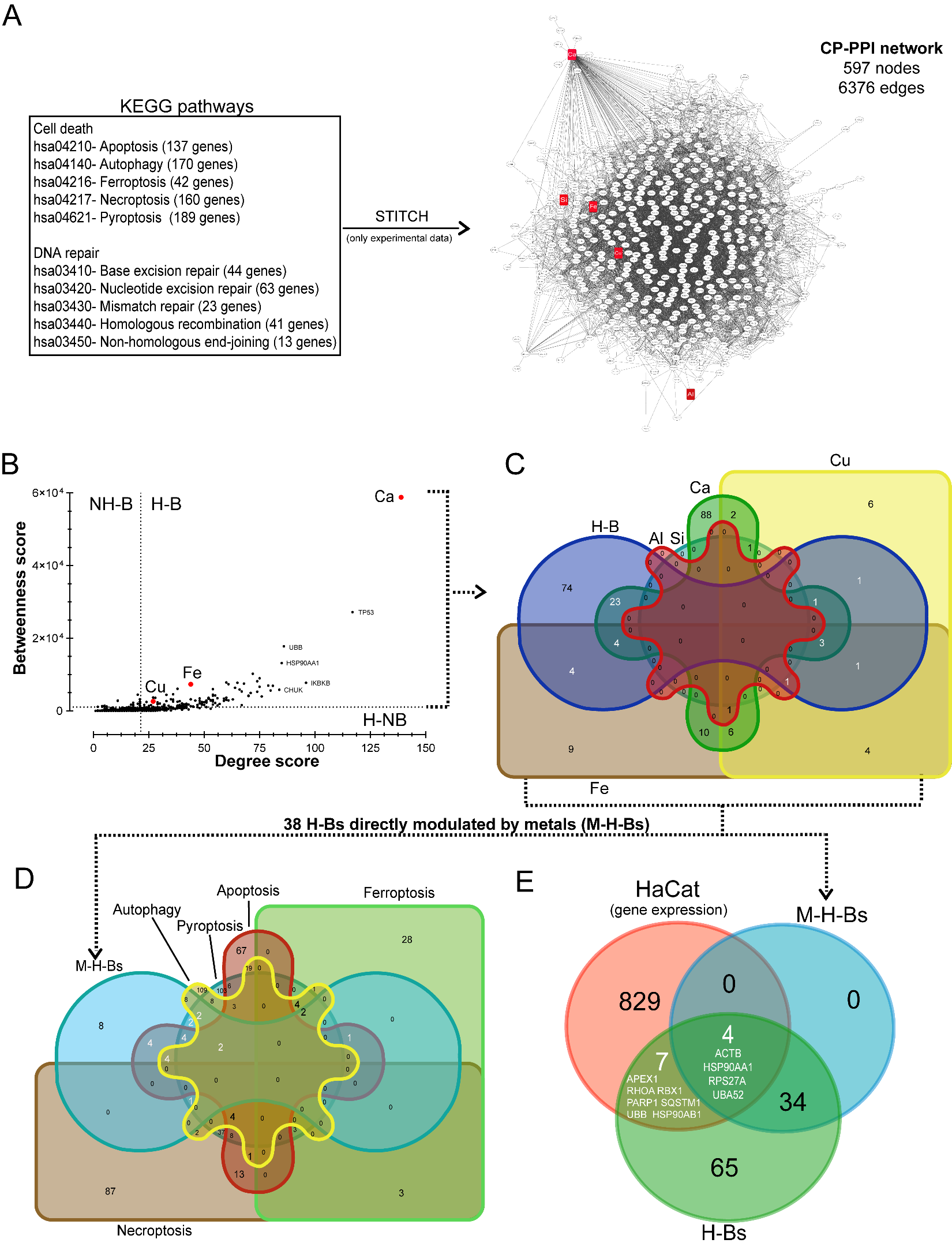


**S3.** Analysis of a chemical-protein (CP) and protein-protein interaction (PPI) network influenced by the metal content in the nanoparticles. (A) A CP-PPI network was predicted using the STITCH platform, considering only experimentally validated data (score = 0.4). KEGG pathways related to various cell death processes were integrated with the metals detected in coal NPs. Nodes highlighted in red represents metals nanoparticles. (B) Centrality analysis of the CP-PPI network. Dashed lines indicate threshold values calculated for each centrality measure, including degree and betweenness. Proteins/genes are represented by black dots, while metal nanoparticles are shown in red. Hubs, identified with the letter "H," represent nodes with degree values exceeding the arithmetic median threshold, while bottlenecks, denoted by the letter "B," are classified based on their respective arithmetic median. (C) A Venn diagram was generated to identify H-B proteins directly modulated by metals, referred to as M-H-Bs. (D) A Venn diagram illustrating the most representative pathways modulated by M-H-Bs. (E) A Venn diagram showing the overlap between constitutively upregulated genes in HaCaT cells, H-B proteins, and M-H-B proteins.

|  |
| --- |

**Table S4.** Data from network centrality analysis. A- Betweeness analysis; B-Degree analysis. Nodes with higher value than arithmetic media are highlighted in yellow. C- H-Bs. D- M-H-Bs.

| **A** |  |  |  | **B** |  |  | **C** |  | **D** |
| --- | --- | --- | --- | --- | --- | --- | --- | --- | --- |
| **Nodes** | **Betweenness** |  |  | **Nodes** | **Degree** |  | **H-Bs** |  | **M-H-Bs** |
| calcium ions | 58823,78736 |  |  | calcium ions | 139 |  | ACTB |  | ACTB |
| TP53 | 27211,42243 |  |  | TP53 | 117 |  | AKT1 |  | NFKBIA |
| UBB | 17823,1765 |  |  | IKBKB | 96 |  | APAF1 |  | FOS |
| HSP90AA1 | 13183,43249 |  |  | UBB | 86 |  | APEX1 |  | ERCC5 |
| BRCA1 | 10242,8854 |  |  | HSP90AA1 | 85 |  | ATM |  | MAPK14 |
| PCNA | 9005,610005 |  |  | CHUK | 84 |  | BAX |  | TP53 |
| CASP3 | 8185,045182 |  |  | TRAF6 | 81 |  | BCL2 |  | PRKACB |
| UBA52 | 7830,955322 |  |  | MAPK1 | 80 |  | BCL2L1 |  | PTEN |
| IKBKB | 7771,522402 |  |  | RELA | 79 |  | BECN1 |  | DDB1 |
| MTOR | 7738,162867 |  |  | RPA1 | 76 |  | BID |  | MAP1LC3A |
| RPS27A | 7516,767378 |  |  | PCNA | 75 |  | BIRC2 |  | RAF1 |
| Fe(III | 7372,368936 |  |  | JAK2 | 75 |  | BLM |  | UBA52 |
| SQSTM1 | 7365,285761 |  |  | CASP3 | 74 |  | BRCA1 |  | IGF1R |
| RELA | 6962,43221 |  |  | TRAF2 | 74 |  | CASP1 |  | MAP3K7 |
| TRAF6 | 6904,427185 |  |  | BRCA1 | 72 |  | CASP3 |  | RAD51 |
| JAK2 | 6656,266512 |  |  | NFKB1 | 71 |  | CASP7 |  | RPS27A |
| PRKDC | 6381,583216 |  |  | JAK1 | 70 |  | CASP8 |  | BLM |
| PYCARD | 6119,697972 |  |  | MAPK3 | 70 |  | CASP9 |  | JUN |
| MAPK14 | 5980,268251 |  |  | RPS27A | 68 |  | CCNH |  | MAPK3 |
| BCL2 | 5880,296997 |  |  | AKT1 | 67 |  | CHUK |  | MAP1LC3B |
| CHUK | 5837,348594 |  |  | ERCC4 | 67 |  | DDB1 |  | PCNA |
| MAPK1 | 5609,506519 |  |  | BCL2 | 66 |  | ERCC2 |  | AKT1 |
| AKT1 | 5368,509052 |  |  | MTOR | 65 |  | ERCC3 |  | PIK3R1 |
| TRAF2 | 5320,621914 |  |  | PRKDC | 64 |  | ERCC4 |  | MAPK1 |
| NFKB1 | 4994,108549 |  |  | RPA2 | 63 |  | ERCC5 |  | HIF1A |
| HIF1A | 4573,475372 |  |  | SQSTM1 | 61 |  | ERCC8 |  | RELA |
| RB1CC1 | 4485,016875 |  |  | PIK3CA | 61 |  | FADD |  | PRKACA |
| ERCC2 | 4481,947462 |  |  | UBA52 | 60 |  | FOS |  | PRKCD |
| PARP1 | 4402,448308 |  |  | CASP8 | 60 |  | Fe |  | NFKB1 |
| PRKACA | 4273,802028 |  |  | IKBKG | 60 |  | GTF2H1 |  | ERCC2 |
| JAK1 | 4231,218671 |  |  | XRCC5 | 59 |  | GTF2H3 |  | HSP90AA1 |
| NFKBIA | 4209,36825 |  |  | MAPK8 | 59 |  | H2AFX |  | CASP3 |
| MAPK3 | 4103,62505 |  |  | XRCC6 | 57 |  | HIF1A |  | PPP2CA |
| RPA1 | 3985,839052 |  |  | PIK3R1 | 56 |  | HMGB1 |  | PIK3CA |
| BECN1 | 3696,329479 |  |  | RIPK1 | 56 |  | HSP90AA1 |  | POLD1 |
| CASP8 | 3676,473789 |  |  | PRKACA | 55 |  | HSP90AB1 |  | TAB1 |
| JUN | 3625,603417 |  |  | JAK3 | 55 |  | IFNB1 |  | ATM |
| MAP1LC3A | 3608,146354 |  |  | MAPK14 | 54 |  | IFNG |  | POLE |
| PIK3R1 | 3520,219968 |  |  | ERCC2 | 54 |  | IGF1R |  |  |
| ATM | 3460,240313 |  |  | NFKBIA | 54 |  | IKBKB |  |  |
| XRCC5 | 3423,810378 |  |  | POLD1 | 54 |  | IKBKG |  |  |
| POLR2A | 3369,838779 |  |  | RAF1 | 54 |  | IRF3 |  |  |
| MAP3K7 | 3334,878005 |  |  | TNFRSF1A | 54 |  | IRF7 |  |  |
| IL8 | 3252,175022 |  |  | TYK2 | 53 |  | IRF9 |  |  |
| HSP90AB1 | 3249,151908 |  |  | STAT1 | 53 |  | JAK1 |  |  |
| BAX | 3233,971364 |  |  | ERCC1 | 53 |  | JAK2 |  |  |
| RPTOR | 3187,397594 |  |  | JUN | 52 |  | JAK3 |  |  |
| RPA2 | 3156,790915 |  |  | POLR2A | 52 |  | JUN |  |  |
| DDB1 | 3129,742689 |  |  | MAP3K7 | 52 |  | MAP1LC3A |  |  |
| IKBKG | 3047,684369 |  |  | PRKCD | 51 |  | MAP1LC3B |  |  |
| POLD1 | 3014,720727 |  |  | ERCC3 | 51 |  | MAP3K5 |  |  |
| TYK2 | 2956,321129 |  |  | MAP3K5 | 51 |  | MAP3K7 |  |  |
| PIK3CA | 2918,451375 |  |  | ERCC8 | 51 |  | MAPK1 |  |  |
| STAT1 | 2878,699844 |  |  | STAT3 | 50 |  | MAPK14 |  |  |
| ATG9A | 2857,36568 |  |  | MSH2 | 50 |  | MAPK3 |  |  |
| PRKCD | 2850,771284 |  |  | PPP2CA | 49 |  | MAPK8 |  |  |
| BCL2L1 | 2834,745412 |  |  | GTF2H1 | 49 |  | MAPK9 |  |  |
| MAPK8 | 2821,069962 |  |  | MNAT1 | 49 |  | MAVS |  |  |
| UVRAG | 2778,85954 |  |  | ATM | 48 |  | MLH1 |  |  |
| RAD51 | 2776,573039 |  |  | RAD51 | 48 |  | MNAT1 |  |  |
| CASP1 | 2758,478873 |  |  | RAD23B | 48 |  | MSH2 |  |  |
| ERCC3 | 2685,00458 |  |  | BIRC3 | 48 |  | MTOR |  |  |
| TP53BP1 | 2678,410145 |  |  | ERCC5 | 47 |  | NFKB1 |  |  |
| copper | 2611,54907 |  |  | RHOA | 47 |  | NFKBIA |  |  |
| TLR3 | 2584,43169 |  |  | RPA3 | 47 |  | OAS3 |  |  |
| RAF1 | 2565,838916 |  |  | BCL2L1 | 46 |  | PARP1 |  |  |
| MAVS | 2554,361544 |  |  | GTF2H3 | 45 |  | PCNA |  |  |
| TAB1 | 2524,377431 |  |  | CCNH | 45 |  | PIK3C3 |  |  |
| TLR4 | 2500,52679 |  |  | Fe(III | 44 |  | PIK3CA |  |  |
| PPP2CA | 2496,612852 |  |  | HSP90AB1 | 44 |  | PIK3R1 |  |  |
| PIK3C3 | 2460,338333 |  |  | RPTOR | 44 |  | POLD1 |  |  |
| STAT3 | 2440,864414 |  |  | BIRC2 | 44 |  | POLE |  |  |
| APEX1 | 2385,851256 |  |  | FOS | 43 |  | POLR2A |  |  |
| MAP3K5 | 2304,367342 |  |  | XPC | 43 |  | POLR2B |  |  |
| ATG7 | 2231,259973 |  |  | PRKACB | 42 |  | PPP2CA |  |  |
| JAK3 | 2206,556528 |  |  | YWHAE | 42 |  | PRKACA |  |  |
| GTF2H1 | 2162,177223 |  |  | TRAF3 | 42 |  | PRKACB |  |  |
| IRF3 | 2092,973532 |  |  | MLH1 | 42 |  | PRKCD |  |  |
| BLM | 2076,191558 |  |  | MRE11A | 42 |  | PRKDC |  |  |
| RAD23B | 2075,336222 |  |  | RIPK2 | 41 |  | PTEN |  |  |
| VDAC1 | 2041,246883 |  |  | IFNG | 41 |  | PYCARD |  |  |
| ACTB | 2038,15939 |  |  | IRS1 | 41 |  | RAD23B |  |  |
| CYLD | 1999,010748 |  |  | BLM | 40 |  | RAD51 |  |  |
| PRKACB | 1996,528695 |  |  | XIAP | 40 |  | RAF1 |  |  |
| YWHAE | 1983,687348 |  |  | FADD | 40 |  | RBX1 |  |  |
| XRCC6 | 1926,359693 |  |  | POLR2B | 40 |  | RELA |  |  |
| MAPK9 | 1871,66755 |  |  | POLE | 40 |  | RHOA |  |  |
| RIPK2 | 1793,967781 |  |  | RAD52 | 40 |  | RIPK1 |  |  |
| TNFRSF1A | 1769,848945 |  |  | TRADD | 40 |  | RIPK2 |  |  |
| APAF1 | 1748,446339 |  |  | MSH6 | 40 |  | RPA1 |  |  |
| FOS | 1739,706521 |  |  | TP53BP1 | 39 |  | RPA2 |  |  |
| MNAT1 | 1733,451665 |  |  | IGF1R | 39 |  | RPS27A |  |  |
| GABARAPL2 | 1685,297158 |  |  | BID | 39 |  | RPTOR |  |  |
| POLB | 1674,488347 |  |  | MAP2K1 | 39 |  | SQSTM1 |  |  |
| IGF1R | 1652,345626 |  |  | HRAS | 39 |  | STAT1 |  |  |
| RAB7A | 1646,537534 |  |  | PYCARD | 38 |  | STAT3 |  |  |
| MSH2 | 1641,906971 |  |  | TAB1 | 38 |  | TAB1 |  |  |
| MAP1LC3B | 1639,562587 |  |  | CASP9 | 38 |  | TNFRSF1A |  |  |
| ERCC5 | 1635,932132 |  |  | H2AFX | 38 |  | TP53 |  |  |
| RBX1 | 1629,660543 |  |  | CASP10 | 38 |  | TP53BP1 |  |  |
| RHOA | 1615,199162 |  |  | PIK3CB | 38 |  | TRAF2 |  |  |
| ERCC4 | 1608,858106 |  |  | PDPK1 | 37 |  | TRAF3 |  |  |
| TRAF3 | 1584,259725 |  |  | PIK3CD | 37 |  | TRAF6 |  |  |
| XIAP | 1547,863925 |  |  | HIF1A | 36 |  | TYK2 |  |  |
| CASP9 | 1515,077321 |  |  | DDB1 | 36 |  | UBA52 |  |  |
| RIPK1 | 1508,944156 |  |  | MAVS | 36 |  | UBB |  |  |
| FADD | 1502,606143 |  |  | FEN1 | 36 |  | ULK1 |  |  |
| CTSS | 1465,301432 |  |  | AKT2 | 36 |  | XIAP |  |  |
| ATG12 | 1455,251943 |  |  | POLR2C | 36 |  | XRCC5 |  |  |
| CASP7 | 1445,18373 |  |  | BAX | 35 |  | XRCC6 |  |  |
| IFNB1 | 1427,417242 |  |  | IRF9 | 35 |  | YWHAE |  |  |
| BID | 1415,467579 |  |  | CSF2RB | 35 |  | Ca |  |  |
| OAS3 | 1365,46022 |  |  | NGF | 35 |  | Cu |  |  |
| POLR2B | 1343,417984 |  |  | MSH3 | 35 |  |  |  |  |
| BIRC2 | 1315,29581 |  |  | POLE2 | 35 |  |  |  |  |
| H2AFX | 1311,679236 |  |  | IFNB1 | 34 |  |  |  |  |
| CTSL1 | 1307,524969 |  |  | XRCC4 | 34 |  |  |  |  |
| FTL | 1267,870132 |  |  | ERCC6 | 34 |  |  |  |  |
| CTSB | 1202,4908 |  |  | PARP1 | 33 |  |  |  |  |
| IFNG | 1199,956466 |  |  | CASP1 | 33 |  |  |  |  |
| GTF2H3 | 1198,737844 |  |  | CFLAR | 33 |  |  |  |  |
| PTEN | 1192,78019 |  |  | POLR2F | 33 |  |  |  |  |
| ATG2B | 1190,945961 |  |  | LIG4 | 33 |  |  |  |  |
| POLG | 1190 |  |  | APAF1 | 32 |  |  |  |  |
| ACSL4 | 1190 |  |  | CASP7 | 32 |  |  |  |  |
| SLC3A2 | 1190 |  |  | SUPT5H | 32 |  |  |  |  |
| DEFB4A | 1190 |  |  | TAB2 | 32 |  |  |  |  |
| CCNH | 1180,18903 |  |  | POLR2E | 32 |  |  |  |  |
| XRCC1 | 1144,127397 |  |  | IFNGR1 | 32 |  |  |  |  |
| HMGB1 | 1140,092337 |  |  | PIK3R3 | 32 |  |  |  |  |
| MLH1 | 1134,711702 |  |  | IRS2 | 32 |  |  |  |  |
| ULK1 | 1116,674391 |  |  | MLH3 | 32 |  |  |  |  |
| IRF9 | 1101,95706 |  |  | PTEN | 31 |  |  |  |  |
| POLE | 1093,681617 |  |  | MAPK11 | 31 |  |  |  |  |
| IRF7 | 1084,589563 |  |  | POLR2H | 31 |  |  |  |  |
| ERCC8 | 1075,46877 |  |  | ACTB | 30 |  |  |  |  |
| BRCC3 | 1047,060384 |  |  | FASLG | 30 |  |  |  |  |
| RPA3 | 1038,055565 |  |  | TNFAIP3 | 30 |  |  |  |  |
| CYCS | 1024,420441 |  |  | CASP2 | 30 |  |  |  |  |
| CAMK2A | 982,7281197 |  |  | RAD50 | 30 |  |  |  |  |
| CASP10 | 979,4359171 |  |  | IFNAR1 | 30 |  |  |  |  |
| ULK2 | 963,0773187 |  |  | BAD | 30 |  |  |  |  |
| BIRC5 | 951,0982783 |  |  | MAP2K2 | 30 |  |  |  |  |
| STIP1 | 915,3342275 |  |  | XPA | 30 |  |  |  |  |
| MAP2K1 | 910,2202395 |  |  | APEX1 | 29 |  |  |  |  |
| WIPI2 | 907,0341519 |  |  | IRF3 | 29 |  |  |  |  |
| FASLG | 894,5542153 |  |  | MAPK9 | 29 |  |  |  |  |
| CSF2RB | 892,4413592 |  |  | IRF7 | 29 |  |  |  |  |
| INS | 891,1625163 |  |  | PRKAA1 | 29 |  |  |  |  |
| EIF2AK2 | 890,6182968 |  |  | RPS3 | 29 |  |  |  |  |
| SUPT5H | 884,51163 |  |  | STAT2 | 29 |  |  |  |  |
| TNFAIP3 | 879,5456489 |  |  | FAS | 29 |  |  |  |  |
| BAK1 | 877,7839125 |  |  | IL3RA | 29 |  |  |  |  |
| CFLAR | 866,7316536 |  |  | POLR2D | 29 |  |  |  |  |
| FEN1 | 864,7129667 |  |  | TRAF1 | 29 |  |  |  |  |
| GZMB | 858,9114721 |  |  | POLR2L | 29 |  |  |  |  |
| HRAS | 857,9129062 |  |  | MAP1LC3A | 28 |  |  |  |  |
| CTSD | 854,5421897 |  |  | EIF2AK2 | 28 |  |  |  |  |
| MRE11A | 854,0025005 |  |  | TOP3A | 28 |  |  |  |  |
| AKT2 | 844,5243733 |  |  | BARD1 | 28 |  |  |  |  |
| NGF | 843,6593505 |  |  | STAT5A | 28 |  |  |  |  |
| GABARAP | 840,5031899 |  |  | RAD51B | 28 |  |  |  |  |
| NLRP3 | 834,5413018 |  |  | TICAM1 | 28 |  |  |  |  |
| YKT6 | 833,6359762 |  |  | AKT3 | 28 |  |  |  |  |
| CAPN1 | 829,4614534 |  |  | POLE4 | 28 |  |  |  |  |
| NOD1 | 822,7136918 |  |  | PMS2 | 28 |  |  |  |  |
| MCL1 | 817,8798768 |  |  | MYD88 | 28 |  |  |  |  |
| PRKAA1 | 814,9267149 |  |  | POLR2K | 28 |  |  |  |  |
| LMNA | 806,0191722 |  |  | POLR2G | 28 |  |  |  |  |
| MAP1LC3C | 804,9354614 |  |  | BECN1 | 27 |  |  |  |  |
| PPID | 803,4542884 |  |  | copper | 27 |  |  |  |  |
| BIRC3 | 795,9978791 |  |  | HMGB1 | 27 |  |  |  |  |
| ATG5 | 778,082047 |  |  | MLST8 | 27 |  |  |  |  |
| MLST8 | 770,2067619 |  |  | RPS6KB1 | 27 |  |  |  |  |
| IRS1 | 767,4798539 |  |  | POLE3 | 27 |  |  |  |  |
| OPTN | 763,9283901 |  |  | POLR2I | 27 |  |  |  |  |
| RPS3 | 759,5248579 |  |  | RBX1 | 26 |  |  |  |  |
| GABARAPL1 | 757,2520308 |  |  | PPP2CB | 26 |  |  |  |  |
| TAB2 | 754,7259153 |  |  | RAD51D | 26 |  |  |  |  |
| TOP3A | 743,1613585 |  |  | PRKACG | 26 |  |  |  |  |
| TUBA4A | 741,4904633 |  |  | MAPK10 | 26 |  |  |  |  |
| RFC1 | 740,3702239 |  |  | IFNAR2 | 26 |  |  |  |  |
| BARD1 | 738,9676225 |  |  | POLR2J | 26 |  |  |  |  |
| CAMK2D | 738,8190158 |  |  | NHEJ1 | 26 |  |  |  |  |
| ATF4 | 733,1605042 |  |  | OAS3 | 25 |  |  |  |  |
| TUBA1A | 725,7593465 |  |  | ULK1 | 25 |  |  |  |  |
| IL1B | 715,9926865 |  |  | IL1B | 25 |  |  |  |  |
| PIK3CB | 714,8392129 |  |  | BRCA2 | 25 |  |  |  |  |
| IL6 | 703,3392077 |  |  | PLCB1 | 25 |  |  |  |  |
| CASP2 | 703,2237446 |  |  | NRAS | 25 |  |  |  |  |
| PPP2CB | 700,2102008 |  |  | PIK3C3 | 24 |  |  |  |  |
| OAS1 | 697,0800341 |  |  | CAMK2A | 24 |  |  |  |  |
| OAS2 | 697,0800341 |  |  | MCL1 | 24 |  |  |  |  |
| STAT5A | 681,8412418 |  |  | ATF4 | 24 |  |  |  |  |
| ATG3 | 680,9485646 |  |  | TSC1 | 24 |  |  |  |  |
| RAD23A | 669,1618979 |  |  | PLCB3 | 24 |  |  |  |  |
| PLA2G4A | 666,7046441 |  |  | TAB3 | 24 |  |  |  |  |
| XRCC4 | 663,6961014 |  |  | NBN | 24 |  |  |  |  |
| POLR2C | 640,6787696 |  |  | DIABLO | 24 |  |  |  |  |
| PDPK1 | 637,0360103 |  |  | PRKCQ | 24 |  |  |  |  |
| AIFM1 | 631,2152252 |  |  | DDB2 | 24 |  |  |  |  |
| ERCC1 | 622,6734428 |  |  | IFNGR2 | 24 |  |  |  |  |
| RAD52 | 606,1844422 |  |  | RFC3 | 24 |  |  |  |  |
| HTRA2 | 597,5754056 |  |  | IFNA14 | 24 |  |  |  |  |
| RAD51B | 581,5301205 |  |  | IFNA4 | 24 |  |  |  |  |
| GLUL | 577,1825081 |  |  | IFNA7 | 24 |  |  |  |  |
| MAP1LC3B2 | 570,3448228 |  |  | IFNA6 | 24 |  |  |  |  |
| ERCC6 | 563,3268384 |  |  | IFNA10 | 24 |  |  |  |  |
| PIK3CD | 561,6558109 |  |  | IFNA21 | 24 |  |  |  |  |
| TSC1 | 558,5852056 |  |  | IFNA5 | 24 |  |  |  |  |
| TFRC | 554,9298603 |  |  | IFNA17 | 24 |  |  |  |  |
| POLR2F | 548,5776586 |  |  | IFNA8 | 24 |  |  |  |  |
| TXN | 546,0151522 |  |  | IFNA16 | 24 |  |  |  |  |
| RIPK3 | 509,3609765 |  |  | IFNA2 | 24 |  |  |  |  |
| IGBP1 | 509,1132009 |  |  | MAP1LC3B | 23 |  |  |  |  |
| UIMC1 | 500,0586495 |  |  | CYCS | 23 |  |  |  |  |
| DNM1L | 498,5614236 |  |  | OAS1 | 23 |  |  |  |  |
| VPS18 | 493,8851195 |  |  | OAS2 | 23 |  |  |  |  |
| RAD51D | 490,4265796 |  |  | RIPK3 | 23 |  |  |  |  |
| STAT2 | 489,4649737 |  |  | CETN2 | 23 |  |  |  |  |
| POLR2E | 486,6014188 |  |  | PLCB2 | 23 |  |  |  |  |
| STAT6 | 482,5769982 |  |  | DCLRE1C | 23 |  |  |  |  |
| FAS | 476,500977 |  |  | RFC4 | 23 |  |  |  |  |
| PLCB3 | 472,5268054 |  |  | RPA4 | 23 |  |  |  |  |
| PRKACG | 470,9666938 |  |  | ATG5 | 22 |  |  |  |  |
| RPS16 | 467,0349525 |  |  | CAMK2D | 22 |  |  |  |  |
| NOD2 | 463,030844 |  |  | TNFRSF10B | 22 |  |  |  |  |
| NLRP1 | 459,7103592 |  |  | RFC5 | 22 |  |  |  |  |
| MAPK11 | 452,9978229 |  |  | TRAF5 | 22 |  |  |  |  |
| NFKBIB | 438,1366118 |  |  | IL8 | 21 |  |  |  |  |
| RAD50 | 434,2700893 |  |  | TLR3 | 21 |  |  |  |  |
| TUBA1B | 433,9786065 |  |  | TLR4 | 21 |  |  |  |  |
| CHMP2B | 433,8961706 |  |  | VDAC1 | 21 |  |  |  |  |
| IFNGR1 | 433,6198387 |  |  | ATG12 | 21 |  |  |  |  |
| XPC | 432,0439271 |  |  | ULK2 | 21 |  |  |  |  |
| PNKP | 431,211635 |  |  | BAK1 | 21 |  |  |  |  |
| GCLM | 429,5081554 |  |  | OPTN | 21 |  |  |  |  |
| RRAGB | 425,1760604 |  |  | RFC1 | 21 |  |  |  |  |
| POLR2H | 424,301167 |  |  | IL6 | 21 |  |  |  |  |
| RAD51C | 421,4650532 |  |  | STAT6 | 21 |  |  |  |  |
| SUGT1 | 415,3851572 |  |  | RPS16 | 21 |  |  |  |  |
| TAB3 | 407,3751014 |  |  | RAD51C | 21 |  |  |  |  |
| TNFRSF10B | 391,9327571 |  |  | CAMK2B | 21 |  |  |  |  |
| IFNAR1 | 388,397476 |  |  | STAT5B | 21 |  |  |  |  |
| VPS4B | 381,5643238 |  |  | TSC2 | 21 |  |  |  |  |
| BRCA2 | 377,7443302 |  |  | RPS15A | 21 |  |  |  |  |
| CUL4A | 377,4497991 |  |  | CDC37 | 21 |  |  |  |  |
| TRADD | 373,8619591 |  |  | BCL2L11 | 21 |  |  |  |  |
| PPIA | 372,2237621 |  |  | MAPK12 | 21 |  |  |  |  |
| PIK3R3 | 370,1131064 |  |  | LIG1 | 21 |  |  |  |  |
| IL3RA | 366,8399995 |  |  | PLCB4 | 21 |  |  |  |  |
| MSH6 | 365,2925791 |  |  | RB1CC1 | 20 |  |  |  |  |
| POLR2D | 363,9990877 |  |  | CYLD | 20 |  |  |  |  |
| TICAM1 | 361,085339 |  |  | ATG3 | 20 |  |  |  |  |
| NBN | 360,5542229 |  |  | HTRA2 | 20 |  |  |  |  |
| BAD | 356,8698037 |  |  | NOD2 | 20 |  |  |  |  |
| DIABLO | 353,1905501 |  |  | CDK7 | 20 |  |  |  |  |
| GADD45A | 348,4631938 |  |  | TOPBP1 | 20 |  |  |  |  |
| ATG16L1 | 348,4172861 |  |  | AKT1S1 | 20 |  |  |  |  |
| CETN2 | 341,5400003 |  |  | MUS81 | 20 |  |  |  |  |
| TF | 329,0725888 |  |  | POLD2 | 20 |  |  |  |  |
| CAMK2B | 328,9607619 |  |  | IL3 | 20 |  |  |  |  |
| CHMP5 | 325,3671745 |  |  | TOP3B | 20 |  |  |  |  |
| BABAM1 | 315,357077 |  |  | GZMB | 19 |  |  |  |  |
| STK11 | 313,9560757 |  |  | GABARAP | 19 |  |  |  |  |
| NTRK1 | 311,4231873 |  |  | TUBA1A | 19 |  |  |  |  |
| STAT5B | 309,4478953 |  |  | NFKBIB | 19 |  |  |  |  |
| POLL | 309,3771984 |  |  | RPS5 | 19 |  |  |  |  |
| PLCB1 | 307,6973224 |  |  | IRS4 | 19 |  |  |  |  |
| IL18 | 304,5968274 |  |  | CAMK2G | 19 |  |  |  |  |
| VPS4A | 298,6952888 |  |  | MAPK13 | 19 |  |  |  |  |
| cAMP | 298,4378424 |  |  | RPS23 | 19 |  |  |  |  |
| EIF2S1 | 297,7970637 |  |  | TANK | 19 |  |  |  |  |
| AKT3 | 295,0031041 |  |  | POLD4 | 19 |  |  |  |  |
| WIPI1 | 293,9231851 |  |  | POLD3 | 19 |  |  |  |  |
| CDK7 | 290,6139849 |  |  | ATG7 | 18 |  |  |  |  |
| MAP2K2 | 286,672022 |  |  | POLB | 18 |  |  |  |  |
| BRIP1 | 283,3307209 |  |  | RAB7A | 18 |  |  |  |  |
| RHEB | 278,1619718 |  |  | XRCC1 | 18 |  |  |  |  |
| ATG13 | 275,3930912 |  |  | MAP1LC3C | 18 |  |  |  |  |
| LIG3 | 275,2988776 |  |  | GABARAPL1 | 18 |  |  |  |  |
| RPS6KB1 | 274,5845214 |  |  | VPS4B | 18 |  |  |  |  |
| USP21 | 272,6825451 |  |  | ATG13 | 18 |  |  |  |  |
| SLC25A5 | 271,6744373 |  |  | APTX | 18 |  |  |  |  |
| PTGES3 | 263,8439431 |  |  | IRAK4 | 18 |  |  |  |  |
| IRS2 | 263,0114856 |  |  | RNF31 | 18 |  |  |  |  |
| PRKCQ | 253,4378023 |  |  | RBBP8 | 18 |  |  |  |  |
| RPS5 | 251,8805621 |  |  | GABARAPL2 | 17 |  |  |  |  |
| VDAC3 | 250,7868528 |  |  | INS | 17 |  |  |  |  |
| VPS39 | 249,3592009 |  |  | CAPN1 | 17 |  |  |  |  |
| IRS4 | 248,2090729 |  |  | TUBA4A | 17 |  |  |  |  |
| PLCB2 | 246,5995696 |  |  | PLA2G4A | 17 |  |  |  |  |
| STAT4 | 245,8234998 |  |  | UIMC1 | 17 |  |  |  |  |
| SPTAN1 | 239,0076743 |  |  | SUGT1 | 17 |  |  |  |  |
| PIK3R4 | 237,3053498 |  |  | NTRK1 | 17 |  |  |  |  |
| STX7 | 234,3636696 |  |  | VPS4A | 17 |  |  |  |  |
| STX17 | 234,3636696 |  |  | BRIP1 | 17 |  |  |  |  |
| SAT1 | 234,2065133 |  |  | CASP6 | 17 |  |  |  |  |
| CAMK2G | 232,162688 |  |  | CASP4 | 17 |  |  |  |  |
| NLRC4 | 226,0364488 |  |  | TNFSF10 | 17 |  |  |  |  |
| VDAC2 | 225,2676918 |  |  | BRCC3 | 16 |  |  |  |  |
| VPS41 | 225,2658868 |  |  | NLRP3 | 16 |  |  |  |  |
| POLE4 | 219,7883558 |  |  | NOD1 | 16 |  |  |  |  |
| TOPBP1 | 218,4252186 |  |  | TXN | 16 |  |  |  |  |
| CASP6 | 216,5493691 |  |  | CHMP2B | 16 |  |  |  |  |
| TSC2 | 211,1021991 |  |  | PNKP | 16 |  |  |  |  |
| RPS15A | 209,2700459 |  |  | CUL4A | 16 |  |  |  |  |
| CDC37 | 208,5656514 |  |  | CHMP5 | 16 |  |  |  |  |
| XPA | 207,4992031 |  |  | EIF2S1 | 16 |  |  |  |  |
| AKT1S1 | 204,9740236 |  |  | RHEB | 16 |  |  |  |  |
| POLE3 | 202,9151127 |  |  | PCBP1 | 16 |  |  |  |  |
| BCL2L11 | 202,8200397 |  |  | CHMP4C | 16 |  |  |  |  |
| MAPK12 | 200,3309509 |  |  | CHMP4B | 16 |  |  |  |  |
| VPS16 | 199,9786982 |  |  | CHMP3 | 16 |  |  |  |  |
| NBR1 | 198,5978308 |  |  | SMPD1 | 16 |  |  |  |  |
| PMS2 | 197,4784504 |  |  | GTF2H2 | 16 |  |  |  |  |
| PCBP2 | 197,3891747 |  |  | BIRC5 | 15 |  |  |  |  |
| UNG | 192,4564107 |  |  | LMNA | 15 |  |  |  |  |
| PCBP1 | 190,6657615 |  |  | RAD23A | 15 |  |  |  |  |
| CP | 190,5151093 |  |  | PTGES3 | 15 |  |  |  |  |
| GPX4 | 190,0485645 |  |  | STAT4 | 15 |  |  |  |  |
| DAPK1 | 188,6909478 |  |  | PCBP2 | 15 |  |  |  |  |
| LIG1 | 188,4362904 |  |  | XRCC3 | 15 |  |  |  |  |
| AMBRA1 | 188,3111128 |  |  | CHMP2A | 15 |  |  |  |  |
| RAB8A | 186,2633128 |  |  | CHMP6 | 15 |  |  |  |  |
| DDB2 | 183,1382043 |  |  | KRAS | 15 |  |  |  |  |
| XRCC3 | 182,4808958 |  |  | ITPR1 | 15 |  |  |  |  |
| MYD88 | 181,1970849 |  |  | RRAS | 15 |  |  |  |  |
| ATG4C | 177,6490117 |  |  | TNFRSF10A | 15 |  |  |  |  |
| GORASP2 | 176,824645 |  |  | PYDC1 | 15 |  |  |  |  |
| RAB1A | 176,0524488 |  |  | PPID | 14 |  |  |  |  |
| CASP4 | 172,1094135 |  |  | IGBP1 | 14 |  |  |  |  |
| CARD8 | 171,5611662 |  |  | NLRP1 | 14 |  |  |  |  |
| LMNB1 | 171,2969483 |  |  | GADD45A | 14 |  |  |  |  |
| PLA2G4B | 167,6026076 |  |  | IL18 | 14 |  |  |  |  |
| MAPK10 | 166,635887 |  |  | NLRC4 | 14 |  |  |  |  |
| VAMP8 | 166,3359255 |  |  | PIK3R2 | 14 |  |  |  |  |
| GSS | 165,8067783 |  |  | BCL2A1 | 14 |  |  |  |  |
| MAPK13 | 160,3144303 |  |  | PKN1 | 14 |  |  |  |  |
| PGAM5 | 158,4427103 |  |  | DEPTOR | 14 |  |  |  |  |
| CHMP4C | 158,3739879 |  |  | TDP1 | 14 |  |  |  |  |
| CHMP4B | 158,3739879 |  |  | RBCK1 | 14 |  |  |  |  |
| CHMP3 | 158,3739879 |  |  | CHMP4A | 14 |  |  |  |  |
| CXCL1 | 157,1584297 |  |  | STIP1 | 13 |  |  |  |  |
| TUBA1C | 157,1382707 |  |  | MAP1LC3B2 | 13 |  |  |  |  |
| APTX | 155,6628917 |  |  | TUBA1B | 13 |  |  |  |  |
| CAPN2 | 151,2053498 |  |  | RRAGB | 13 |  |  |  |  |
| TRIP6 | 148,1701092 |  |  | STK11 | 13 |  |  |  |  |
| LIG4 | 144,2226421 |  |  | POLL | 13 |  |  |  |  |
| CCL5 | 142,2374292 |  |  | LIG3 | 13 |  |  |  |  |
| TRAF1 | 139,6442609 |  |  | CASR | 13 |  |  |  |  |
| CHMP2A | 138,4883543 |  |  | ATG4B | 13 |  |  |  |  |
| CHMP6 | 138,4883543 |  |  | ITPR3 | 13 |  |  |  |  |
| LAMP2 | 138,1288496 |  |  | ZBP1 | 13 |  |  |  |  |
| SH3GLB1 | 137,0393291 |  |  | RPS13 | 13 |  |  |  |  |
| RPS23 | 136,3843434 |  |  | CHMP7 | 13 |  |  |  |  |
| PLCB4 | 135,7862634 |  |  | AIFM1 | 12 |  |  |  |  |
| MLH3 | 132,6358422 |  |  | ATG16L1 | 12 |  |  |  |  |
| PRKAA2 | 132,3080475 |  |  | DAPK1 | 12 |  |  |  |  |
| GBP2 | 131,2875667 |  |  | PRKAA2 | 12 |  |  |  |  |
| DCLRE1C | 130,8485595 |  |  | IL1A | 12 |  |  |  |  |
| MUS81 | 130,7151584 |  |  | RAD54B | 12 |  |  |  |  |
| POLR2K | 130,3074292 |  |  | DAB2IP | 12 |  |  |  |  |
| POLR2L | 129,5645986 |  |  | RAD54L | 12 |  |  |  |  |
| PIK3R2 | 127,2247884 |  |  | POLR2J3 | 12 |  |  |  |  |
| MSH3 | 127,0908813 |  |  | POLR2J2 | 12 |  |  |  |  |
| TXN2 | 126,3088612 |  |  | ATG9A | 11 |  |  |  |  |
| OGG1 | 126,2229853 |  |  | UVRAG | 11 |  |  |  |  |
| TANK | 124,5532105 |  |  | PPIA | 11 |  |  |  |  |
| CCL2 | 122,78789 |  |  | cAMP | 11 |  |  |  |  |
| FTH1 | 122,4208719 |  |  | SLC25A5 | 11 |  |  |  |  |
| ANTXR2 | 112,6282941 |  |  | SPTAN1 | 11 |  |  |  |  |
| IFNGR2 | 112,5833026 |  |  | AMBRA1 | 11 |  |  |  |  |
| POLE2 | 111,9831591 |  |  | RAB8A | 11 |  |  |  |  |
| CYBB | 108,8884405 |  |  | LMNB1 | 11 |  |  |  |  |
| IFNAR2 | 108,2187429 |  |  | CCL5 | 11 |  |  |  |  |
| NLRP12 | 106,3678713 |  |  | GBP2 | 11 |  |  |  |  |
| POLR2G | 105,070969 |  |  | CHMP1A | 11 |  |  |  |  |
| CASR | 100,6155239 |  |  | CASP12 | 11 |  |  |  |  |
| POLD2 | 99,59896476 |  |  | SHARPIN | 11 |  |  |  |  |
| PYGL | 98,5916169 |  |  | POLM | 11 |  |  |  |  |
| TAX1BP1 | 97,19261334 |  |  | ITPR2 | 11 |  |  |  |  |
| PTPN13 | 95,14008995 |  |  | C12orf44 | 11 |  |  |  |  |
| KRAS | 94,60291304 |  |  | ATG4A | 11 |  |  |  |  |
| ITPR1 | 93,8790313 |  |  | RPS6KB2 | 11 |  |  |  |  |
| IL3 | 92,26313428 |  |  | RFC2 | 11 |  |  |  |  |
| SMPD1 | 91,87922204 |  |  | TFRC | 10 |  |  |  |  |
| NRAS | 90,79872808 |  |  | NBR1 | 10 |  |  |  |  |
| IL1A | 90,71448566 |  |  | CARD8 | 10 |  |  |  |  |
| GLUD1 | 89,31510698 |  |  | CAPN2 | 10 |  |  |  |  |
| GLUD2 | 89,31510698 |  |  | OGG1 | 10 |  |  |  |  |
| SSBP1 | 88,35170344 |  |  | TAX1BP1 | 10 |  |  |  |  |
| CALCOCO2 | 88,19873396 |  |  | PTPN13 | 10 |  |  |  |  |
| RAD54B | 86,01117066 |  |  | TICAM2 | 10 |  |  |  |  |
| ACTG1 | 83,00170793 |  |  | BIRC6 | 10 |  |  |  |  |
| BCL2A1 | 81,4586953 |  |  | RRAGC | 10 |  |  |  |  |
| AIM2 | 80,76689317 |  |  | CUL4B | 10 |  |  |  |  |
| IRAK4 | 78,11584699 |  |  | PALB2 | 10 |  |  |  |  |
| ALOX15 | 77,94456644 |  |  | BBC3 | 10 |  |  |  |  |
| ATG4B | 77,70329885 |  |  | PMAIP1 | 10 |  |  |  |  |
| TOP3B | 76,22048836 |  |  | IFNA1 | 10 |  |  |  |  |
| TICAM2 | 74,68722461 |  |  | CHMP1B | 10 |  |  |  |  |
| PKN1 | 73,79466353 |  |  | WIPI2 | 9 |  |  |  |  |
| DEPTOR | 73,54414486 |  |  | VPS18 | 9 |  |  |  |  |
| SNAP29 | 72,7862911 |  |  | TF | 9 |  |  |  |  |
| DAB2IP | 72,66419659 |  |  | BABAM1 | 9 |  |  |  |  |
| POLR2I | 71,97269149 |  |  | VDAC3 | 9 |  |  |  |  |
| BIRC6 | 71,34550052 |  |  | VPS39 | 9 |  |  |  |  |
| PRF1 | 71,10725651 |  |  | STX7 | 9 |  |  |  |  |
| CAMKK2 | 70,49400665 |  |  | STX17 | 9 |  |  |  |  |
| RFC4 | 69,1148717 |  |  | VDAC2 | 9 |  |  |  |  |
| RPA4 | 68,5560332 |  |  | VPS41 | 9 |  |  |  |  |
| ATG14 | 66,94573289 |  |  | VPS16 | 9 |  |  |  |  |
| ITPR3 | 65,89194545 |  |  | ATG4C | 9 |  |  |  |  |
| DAPK3 | 63,92802691 |  |  | PGAM5 | 9 |  |  |  |  |
| RRAGC | 63,78663617 |  |  | TUBA1C | 9 |  |  |  |  |
| GTF2H2 | 60,48941606 |  |  | CCL2 | 9 |  |  |  |  |
| WDFY3 | 60,27659079 |  |  | CALCOCO2 | 9 |  |  |  |  |
| DAXX | 60,13085874 |  |  | ALOX15 | 9 |  |  |  |  |
| PANX1 | 59,27759541 |  |  | ERBB2IP | 9 |  |  |  |  |
| GADD45B | 56,70017327 |  |  | RRAGA | 9 |  |  |  |  |
| CYBA | 54,55661717 |  |  | DDIT3 | 9 |  |  |  |  |
| CHMP1A | 52,5775329 |  |  | GTF2H4 | 9 |  |  |  |  |
| VPS33A | 52,50275117 |  |  | IFNA13 | 9 |  |  |  |  |
| ANTXR1 | 50,92159885 |  |  | YKT6 | 8 |  |  |  |  |
| RRAS | 50,86795343 |  |  | GLUL | 8 |  |  |  |  |
| RFC3 | 47,14026929 |  |  | PLA2G4B | 8 |  |  |  |  |
| BNIP3 | 46,70967183 |  |  | CXCL1 | 8 |  |  |  |  |
| SLC25A4 | 45,35241569 |  |  | TRIP6 | 8 |  |  |  |  |
| CASP12 | 45,05153273 |  |  | DAXX | 8 |  |  |  |  |
| TDP1 | 44,76806021 |  |  | ERN1 | 8 |  |  |  |  |
| CUL4B | 43,73909541 |  |  | DNTT | 8 |  |  |  |  |
| POLR2J | 42,806607 |  |  | ATG4D | 8 |  |  |  |  |
| TNFSF10 | 40,43886533 |  |  | GPRC6A | 8 |  |  |  |  |
| MFN2 | 39,86130384 |  |  | RNASEL | 8 |  |  |  |  |
| RFC5 | 38,91336887 |  |  | RRAGD | 8 |  |  |  |  |
| GORASP1 | 38,87532822 |  |  | USP21 | 7 |  |  |  |  |
| PALB2 | 38,74936215 |  |  | UNG | 7 |  |  |  |  |
| ERBB2IP | 37,98699905 |  |  | CP | 7 |  |  |  |  |
| TXNIP | 37,27858388 |  |  | FTH1 | 7 |  |  |  |  |
| GADD45G | 34,71567509 |  |  | ACTG1 | 7 |  |  |  |  |
| TRAF5 | 33,8026138 |  |  | AIM2 | 7 |  |  |  |  |
| RNF31 | 33,25945213 |  |  | WDFY3 | 7 |  |  |  |  |
| H2AFY | 33,2309441 |  |  | GADD45B | 7 |  |  |  |  |
| CTSK | 32,89396551 |  |  | VPS33A | 7 |  |  |  |  |
| RAD54L | 32,80477728 |  |  | DDIT4 | 7 |  |  |  |  |
| GCLC | 32,18246231 |  |  | EIF2AK4 | 7 |  |  |  |  |
| RBCK1 | 31,13628713 |  |  | RRAS2 | 7 |  |  |  |  |
| DDIT4 | 30,71391453 |  |  | MUTYH | 7 |  |  |  |  |
| NHEJ1 | 29,50877744 |  |  | GBP1 | 7 |  |  |  |  |
| POLD4 | 29,41530299 |  |  | CTSB | 6 |  |  |  |  |
| POLD3 | 29,41530299 |  |  | CTSD | 6 |  |  |  |  |
| LAMP1 | 29,26174768 |  |  | GCLM | 6 |  |  |  |  |
| PSTPIP1 | 28,97255543 |  |  | LAMP2 | 6 |  |  |  |  |
| TUBA3E | 28,35525448 |  |  | TXN2 | 6 |  |  |  |  |
| SHARPIN | 27,70715361 |  |  | SNAP29 | 6 |  |  |  |  |
| ERN1 | 27,50724238 |  |  | CAMKK2 | 6 |  |  |  |  |
| RBBP8 | 27,45179615 |  |  | PANX1 | 6 |  |  |  |  |
| TUBA3C | 26,18141451 |  |  | CYBA | 6 |  |  |  |  |
| POLM | 25,61859832 |  |  | SLC25A4 | 6 |  |  |  |  |
| STEAP3 | 25,6015545 |  |  | GADD45G | 6 |  |  |  |  |
| ENDOG | 25,49075099 |  |  | GCLC | 6 |  |  |  |  |
| PRNP | 25,37619843 |  |  | PKN2 | 6 |  |  |  |  |
| ITPR2 | 23,67282649 |  |  | EIF2AK3 | 6 |  |  |  |  |
| TNFRSF10A | 21,82417238 |  |  | TDG | 6 |  |  |  |  |
| EIF2AK4 | 21,651213 |  |  | HMOX1 | 6 |  |  |  |  |
| ZBP1 | 21,44068448 |  |  | HRK | 6 |  |  |  |  |
| C12orf44 | 20,940244 |  |  | NTHL1 | 6 |  |  |  |  |
| PARP2 | 19,71774292 |  |  | ATG10 | 6 |  |  |  |  |
| CHMP4A | 18,84421867 |  |  | GTF2H5 | 6 |  |  |  |  |
| SLC25A6 | 17,45050747 |  |  | GTF2H2C | 6 |  |  |  |  |
| P2RX7 | 16,68553871 |  |  | XRCC2 | 6 |  |  |  |  |
| PKN2 | 15,74305846 |  |  | CXCL2 | 6 |  |  |  |  |
| RPS13 | 14,73290784 |  |  | CXCL3 | 6 |  |  |  |  |
| RRAGA | 13,51036558 |  |  | CTSS | 5 |  |  |  |  |
| EIF2AK3 | 13,2032123 |  |  | FTL | 5 |  |  |  |  |
| MFN1 | 13,17649005 |  |  | ATG2B | 5 |  |  |  |  |
| DNTT | 12,54757389 |  |  | DNM1L | 5 |  |  |  |  |
| SPATA2 | 12,18220722 |  |  | WIPI1 | 5 |  |  |  |  |
| DDIT3 | 12,06329189 |  |  | PIK3R4 | 5 |  |  |  |  |
| TNF | 11,46655137 |  |  | SAT1 | 5 |  |  |  |  |
| NAIP | 11,37845869 |  |  | GORASP2 | 5 |  |  |  |  |
| ATG4A | 11,09047093 |  |  | RAB1A | 5 |  |  |  |  |
| TRPM2 | 10,7808891 |  |  | VAMP8 | 5 |  |  |  |  |
| TDG | 10,23297421 |  |  | GSS | 5 |  |  |  |  |
| IL33 | 8,672344637 |  |  | SH3GLB1 | 5 |  |  |  |  |
| DFFB | 8,435224766 |  |  | PYGL | 5 |  |  |  |  |
| EME1 | 8,417373696 |  |  | GLUD1 | 5 |  |  |  |  |
| RPS6KB2 | 7,752001694 |  |  | GLUD2 | 5 |  |  |  |  |
| DFFA | 7,516622126 |  |  | SSBP1 | 5 |  |  |  |  |
| ATG4D | 7,305357878 |  |  | DAPK3 | 5 |  |  |  |  |
| RRAS2 | 6,735822291 |  |  | BNIP3 | 5 |  |  |  |  |
| DAPK2 | 6,553030257 |  |  | MFN2 | 5 |  |  |  |  |
| MBD4 | 6,346096642 |  |  | H2AFY | 5 |  |  |  |  |
| LMNB2 | 6,21670426 |  |  | P2RX7 | 5 |  |  |  |  |
| HMOX1 | 6,064751366 |  |  | NAIP | 5 |  |  |  |  |
| IFNA14 | 5,833446821 |  |  | LMNB2 | 5 |  |  |  |  |
| IFNA4 | 5,833446821 |  |  | SLC40A1 | 5 |  |  |  |  |
| IFNA7 | 5,833446821 |  |  | NLRX1 | 5 |  |  |  |  |
| IFNA6 | 5,833446821 |  |  | MRAS | 5 |  |  |  |  |
| IFNA10 | 5,833446821 |  |  | UVSSA | 5 |  |  |  |  |
| IFNA21 | 5,833446821 |  |  | CTSL1 | 4 |  |  |  |  |
| IFNA5 | 5,833446821 |  |  | POLG | 4 |  |  |  |  |
| IFNA17 | 5,833446821 |  |  | GPX4 | 4 |  |  |  |  |
| IFNA8 | 5,833446821 |  |  | ATG14 | 4 |  |  |  |  |
| IFNA16 | 5,833446821 |  |  | TXNIP | 4 |  |  |  |  |
| IFNA2 | 5,833446821 |  |  | LAMP1 | 4 |  |  |  |  |
| BBC3 | 5,668196904 |  |  | STEAP3 | 4 |  |  |  |  |
| PMAIP1 | 5,668196904 |  |  | PRNP | 4 |  |  |  |  |
| ATG16L2 | 5,655597694 |  |  | PARP2 | 4 |  |  |  |  |
| GTF2H4 | 5,492702586 |  |  | SLC25A6 | 4 |  |  |  |  |
| RFC2 | 5,429524188 |  |  | MFN1 | 4 |  |  |  |  |
| PLA2G4D | 4,991995965 |  |  | TNF | 4 |  |  |  |  |
| PLA2G4F | 4,991995965 |  |  | EME1 | 4 |  |  |  |  |
| PLA2G4C | 4,991995965 |  |  | DFFA | 4 |  |  |  |  |
| PLA2G4E | 4,991995965 |  |  | PLA2G4D | 4 |  |  |  |  |
| HRK | 4,743162658 |  |  | PLA2G4F | 4 |  |  |  |  |
| CARD6 | 4,081223642 |  |  | PLA2G4C | 4 |  |  |  |  |
| MUTYH | 3,973765983 |  |  | PLA2G4E | 4 |  |  |  |  |
| DHX33 | 3,666634684 |  |  | CARD6 | 4 |  |  |  |  |
| MPG | 3,596168409 |  |  | IKBKE | 4 |  |  |  |  |
| RAB33B | 3,48653714 |  |  | CARD16 | 4 |  |  |  |  |
| CTSL2 | 3,416535485 |  |  | SYCP3 | 4 |  |  |  |  |
| SHFM1 | 3,407436112 |  |  | ATG2A | 4 |  |  |  |  |
| IFNA1 | 3,215723063 |  |  | CASP5 | 4 |  |  |  |  |
| GPRC6A | 2,285062326 |  |  | TRPM7 | 4 |  |  |  |  |
| PYDC2 | 2,045512821 |  |  | TUBA8 | 4 |  |  |  |  |
| NTHL1 | 1,985895568 |  |  | SLC11A2 | 4 |  |  |  |  |
| IKBKE | 1,86539219 |  |  | NLRP12 | 3 |  |  |  |  |
| CARD16 | 1,790437068 |  |  | PRF1 | 3 |  |  |  |  |
| ATG10 | 1,587955166 |  |  | ANTXR1 | 3 |  |  |  |  |
| MLKL | 1,470073277 |  |  | GORASP1 | 3 |  |  |  |  |
| SYCP3 | 1,185702503 |  |  | PSTPIP1 | 3 |  |  |  |  |
| CARD9 | 1,154432273 |  |  | TUBA3E | 3 |  |  |  |  |
| IFNA13 | 1,065271974 |  |  | TUBA3C | 3 |  |  |  |  |
| ATG2A | 0,945960957 |  |  | ENDOG | 3 |  |  |  |  |
| CARD18 | 0,88654747 |  |  | SPATA2 | 3 |  |  |  |  |
| CASP5 | 0,88654747 |  |  | TRPM2 | 3 |  |  |  |  |
| SLC40A1 | 0,785714286 |  |  | DFFB | 3 |  |  |  |  |
| MTMR14 | 0,489415389 |  |  | DAPK2 | 3 |  |  |  |  |
| GTF2H5 | 0,314516129 |  |  | MPG | 3 |  |  |  |  |
| GTF2H2C | 0,314516129 |  |  | SHFM1 | 3 |  |  |  |  |
| XRCC2 | 0,111111111 |  |  | PYDC2 | 3 |  |  |  |  |
| CHMP1B | 0 |  |  | MLKL | 3 |  |  |  |  |
| PARP4 | 0 |  |  | CARD9 | 3 |  |  |  |  |
| CXCL2 | 0 |  |  | CARD18 | 3 |  |  |  |  |
| CTSC | 0 |  |  | NEIL1 | 3 |  |  |  |  |
| POLG2 | 0 |  |  | SLC25A31 | 3 |  |  |  |  |
| FAM48A | 0 |  |  | IFI16 | 3 |  |  |  |  |
| FTMT | 0 |  |  | ACSL4 | 2 |  |  |  |  |
| H2AFY2 | 0 |  |  | SLC3A2 | 2 |  |  |  |  |
| NLRX1 | 0 |  |  | DEFB4A | 2 |  |  |  |  |
| CXCL3 | 0 |  |  | ANTXR2 | 2 |  |  |  |  |
| DEFB4B | 0 |  |  | CYBB | 2 |  |  |  |  |
| WDR45 | 0 |  |  | CTSK | 2 |  |  |  |  |
| MRAS | 0 |  |  | IL33 | 2 |  |  |  |  |
| UVSSA | 0 |  |  | MBD4 | 2 |  |  |  |  |
| TRPV2 | 0 |  |  | ATG16L2 | 2 |  |  |  |  |
| H2AFJ | 0 |  |  | DHX33 | 2 |  |  |  |  |
| SPATA2L | 0 |  |  | RAB33B | 2 |  |  |  |  |
| TUBA3D | 0 |  |  | CTSL2 | 2 |  |  |  |  |
| CHMP7 | 0 |  |  | MTMR14 | 2 |  |  |  |  |
| NEIL1 | 0 |  |  | PARP4 | 2 |  |  |  |  |
| SLC39A8 | 0 |  |  | FAM48A | 2 |  |  |  |  |
| RNASEL | 0 |  |  | H2AFY2 | 2 |  |  |  |  |
| ZFYVE1 | 0 |  |  | PARG | 2 |  |  |  |  |
| GBP1 | 0 |  |  | TP53AIP1 | 2 |  |  |  |  |
| SLC25A31 | 0 |  |  | SAT2 | 2 |  |  |  |  |
| TRPM7 | 0 |  |  | aluminum | 2 |  |  |  |  |
| TUBA8 | 0 |  |  | NRBF2 | 2 |  |  |  |  |
| PARG | 0 |  |  | silicon | 2 |  |  |  |  |
| PYGM | 0 |  |  | MEFV | 2 |  |  |  |  |
| SLC7A11 | 0 |  |  | SMUG1 | 2 |  |  |  |  |
| TP53AIP1 | 0 |  |  | PYGB | 2 |  |  |  |  |
| IFI16 | 0 |  |  | CTSC | 1 |  |  |  |  |
| PARP3 | 0 |  |  | POLG2 | 1 |  |  |  |  |
| TP53INP2 | 0 |  |  | FTMT | 1 |  |  |  |  |
| SAT2 | 0 |  |  | DEFB4B | 1 |  |  |  |  |
| SMCR8 | 0 |  |  | WDR45 | 1 |  |  |  |  |
| ACSL3 | 0 |  |  | TRPV2 | 1 |  |  |  |  |
| aluminum | 0 |  |  | H2AFJ | 1 |  |  |  |  |
| POLR2J3 | 0 |  |  | SPATA2L | 1 |  |  |  |  |
| NRBF2 | 0 |  |  | TUBA3D | 1 |  |  |  |  |
| PYDC1 | 0 |  |  | SLC39A8 | 1 |  |  |  |  |
| NAMPT | 0 |  |  | ZFYVE1 | 1 |  |  |  |  |
| LPCAT3 | 0 |  |  | PYGM | 1 |  |  |  |  |
| WDR41 | 0 |  |  | SLC7A11 | 1 |  |  |  |  |
| SLC11A2 | 0 |  |  | PARP3 | 1 |  |  |  |  |
| silicon | 0 |  |  | TP53INP2 | 1 |  |  |  |  |
| TUBAL3 | 0 |  |  | SMCR8 | 1 |  |  |  |  |
| CTSF | 0 |  |  | ACSL3 | 1 |  |  |  |  |
| MEFV | 0 |  |  | NAMPT | 1 |  |  |  |  |
| RRAGD | 0 |  |  | LPCAT3 | 1 |  |  |  |  |
| C9orf72 | 0 |  |  | WDR41 | 1 |  |  |  |  |
| POLR2J2 | 0 |  |  | TUBAL3 | 1 |  |  |  |  |
| SMUG1 | 0 |  |  | CTSF | 1 |  |  |  |  |
| PYGB | 0 |  |  | C9orf72 | 1 |  |  |  |  |
|  | 1043,688442 |  |  |  | 21,36013 |  |  |  |  |
|  |  |  |  |  |  |  |  |  |  |
